# Elucidation of TRIM25 ubiquitination targets involved in diverse cellular and antiviral processes

**DOI:** 10.1371/journal.ppat.1010743

**Published:** 2022-09-06

**Authors:** Emily Yang, Serina Huang, Yasaman Jami-Alahmadi, Gerald M. McInerney, James A. Wohlschlegel, Melody M. H. Li

**Affiliations:** 1 Molecular Biology Institute, University of California, Los Angeles, California, United States of America; 2 Department of Microbiology, Immunology and Molecular Genetics, University of California, Los Angeles, California, United States of America; 3 Department of Human Genetics, David Geffen School of Medicine, University of California, Los Angeles, California, United States of America; 4 Department of Biological Chemistry, David Geffen School of Medicine, University of California, Los Angeles, California, United States of America; 5 Department of Microbiology, Tumor, and Cell Biology, Karolinska Institutet, Stockholm, Sweden; 6 AIDS Institute, David Geffen School of Medicine, University of California, Los Angeles, California, United States of America; University of North Carolina at Chapel Hill, UNITED STATES

## Abstract

The tripartite motif (TRIM) family of E3 ubiquitin ligases is well known for its roles in antiviral restriction and innate immunity regulation, in addition to many other cellular pathways. In particular, TRIM25-mediated ubiquitination affects both carcinogenesis and antiviral response. While individual substrates have been identified for TRIM25, it remains unclear how it regulates diverse processes. Here we characterized a mutation, R54P, critical for TRIM25 catalytic activity, which we successfully utilized to “trap” substrates. We demonstrated that TRIM25 targets proteins implicated in stress granule formation (G3BP1/2), nonsense-mediated mRNA decay (UPF1), nucleoside synthesis (NME1), and mRNA translation and stability (PABPC4). The R54P mutation abolishes TRIM25 inhibition of alphaviruses independently of the host interferon response, suggesting that this antiviral effect is a direct consequence of ubiquitination. Consistent with that, we observed diminished antiviral activity upon knockdown of several TRIM25-R54P specific interactors including NME1 and PABPC4. Our findings highlight that multiple substrates mediate the cellular and antiviral activities of TRIM25, illustrating the multi-faceted role of this ubiquitination network in modulating diverse biological processes.

## Author summary

Ubiquitin E3 ligases each interact with and ubiquitinate a subset of cellular proteins, thereby regulating specific cellular processes. Tripartite motif containing protein 25 (TRIM25) is one such E3 ligase involved in carcinogenesis and antiviral innate immunity. TRIM25 catalytic activity is indispensable for the host antiviral response against alphaviruses, an arthropod-borne group of RNA viruses possessing expanding distributions and pandemic potential. However, it remains poorly understood which TRIM25 substrates mediate viral inhibition. To complicate the matter, identification of E3 ligase substrates is technically challenging, given the transient nature of ligase-substrate interactions. Here, we present the first comprehensive ubiquitinome study utilizing a novel “substrate-trapping” approach to identify TRIM25 target proteins. We found that TRIM25 ubiquitinates key players in translational and nucleic acid metabolic processes, specifically involving stress granule formation, nonsense-mediated mRNA decay, nucleotide synthesis, and translation initiation. In addition, TRIM25 ligase activity is critical for its inhibition of diverse alphaviruses through viral translation suppression, highlighting the importance of ubiquitination in driving antiviral activity in this context. Our study both provides new insights into understanding the innate immune and cell biology roles of TRIM25 and paves the way forward for identification of novel TRIM substrates at large.

## Introduction

Addition of ubiquitin, or ubiquitination, is a post-translational modification that is highly conserved in eukaryotic organisms, and operates in myriad cellular pathways. Ubiquitin is a small, 76 amino acid protein that must be activated by E1 enzymes, passed to E2 carrier enzymes, and finally covalently attached to lysines on substrates by E3 ligases. Though only one enzyme is needed at each step, their numbers vary widely. Humans encode 2 E1 enzymes, about 40 E2 enzymes, and upwards of 600 E3 ligases [1,2]. This vast number of E3 ligases is needed because they determine substrate specificity; however, the means by which E3 ligases identify their substrates and the array of substrates ubiquitinated by any given E3 ligase remain largely unknown.

The tripartite motif containing protein (TRIM) family is one of the largest families of E3 ligases, with over 70 *TRIM* genes in humans [3]. TRIMs share three common domains at their N-terminus–the catalytic RING domain, 1 to 2 B-Box domains, and a coiled-coil domain–but differ in their C-termini [3]. These varied C-termini determine TRIM substrate specificity, allowing this large family of proteins to regulate diverse cellular processes, including but not limited to viral restriction, immune signaling, stress responses, proliferation, and differentiation [4–7]. Mutations in TRIM genes have been associated with rare genetic diseases, including developmental, muscular, and neurological disorders [8,9]. However, development of targeted therapeutic approaches has been hindered by not only the lack of knowledge on their specific substrates, but also the frequent involvement of TRIMs in multiple cellular processes. One prime example is TRIM25, which functions in both cancer and antiviral innate immunity [10,11]. When examined in the context of cancer, TRIM25-mediated ubiquitination primarily targets varied proteins for proteolytic degradation, which can either enhance or hinder carcinogenesis [12–16].

Many of the TRIM proteins are upregulated by interferon (IFN) and play significant roles in the host innate immune response [7]. Upon detection of viral infection by the host cell, type I IFN is produced, inducing expression of hundreds of IFN-stimulated genes (ISGs) to establish an antiviral environment [17,18]. TRIM25 is one such ISG which not only stimulates innate immune signaling by ubiquitinating and activating the dsRNA sensor RIG-I, but also functions as a critical co-factor of another ISG, zinc finger antiviral protein (ZAP) [19–21]. While TRIM25 has been shown to complex with ZAP in the context of several different viral infections [22], its ligase activity has only been tied to its participation in blocking translation of incoming RNA genomes of alphavirus (family Togaviridae) [20]. Given that ubiquitination of ZAP or lack thereof fails to affect its viral translation inhibition [20], it is likely that TRIM25 antiviral involvement depends on its ubiquitination of other cellular proteins. Interestingly, both TRIM25 and ZAP not only bind viral RNA but also interact with other RNA binding proteins, implying that proteins involved in RNA processes may feature prominently among TRIM25 substrates [23–26].

In light of this question, we set out to identify novel TRIM25 substrates that may play a role in translation and RNA processes. Because identification of E3 ligase substrates is technically challenging due to the transient nature of ligase-substrate interactions, we utilized a “substrate trapping” approach similar to previously reported [27] to capture TRIM25 interactors in a co-immunoprecipitation (IP)/mass spectrometry (MS) experiment. We sought to generate a TRIM25 mutant that would be unable to interact with the upstream E2 carrier enzyme, thus simultaneously rendering it incapable of ubiquitination and prolonging its interactions with substrates. We identified a point mutation, R54P, in the TRIM25 RING catalytic domain, which almost completely abolishes its autoubiquitination in cells.

While almost all of the more highly enriched interactors are shared by both TRIM25-WT and -R54P, we found that TRIM25-R54P enriches for additional interactors as compared to TRIM25-WT. Further characterization of some of the most highly enriched interactors, Ras-GTPase-activating protein SH3-domain binding proteins (G3BP) 1 and 2, RNA helicase up-frameshift protein 1 (UPF1), nucleoside diphosphate kinase 1 (NME1), and poly-adenylate binding protein cytoplasmic 4 (PABPC4), has validated their identification as novel TRIM25 substrates. We identified NME1 and PABPC4 as TRIM25-R54P-specific interactors during viral infection. Moreover, upon characterization of its antiviral activity, the TRIM25-R54P mutant demonstrates a complete loss of inhibition against a panel of Old World and New World alphaviruses albeit higher IFN and ISG expression compared to WT, suggesting that ubiquitination of TRIM25 substrates directly leads to activation of an antiviral state. Altogether, we have identified both known and novel interactors as TRIM25 substrates, and demonstrated the validity of this “substrate trapping” approach in identifying bona fide E3 ligase substrates. We have shed light on the ways that TRIM25-mediated ubiquitination might target substrates to modulate translation, nucleic acid metabolism, and antiviral response, paving the way for further work characterizing the critical role of TRIMs in diverse cellular and viral processes.

## Results

### Point mutations in TRIM25 RING domain almost completely abolish TRIM25 auto-polyubiquitination

It is technically challenging to identify E3 ligase-substrate interactions as they are often transient, resulting in proteasomal degradation or a change in localization or activity of the substrates. In order to enrich for transient E3 ligase-substrate interactions, we turned to a less conventional co-IP approach that makes use of E3 mutants unable to interact with E2 conjugating enzymes. This prevents ubiquitin transfer to E3 substrates and their subsequent targeting to other cellular pathways and as a result, “trapping” these substrates. This approach successfully identified the cellular ‘structural maintenance of chromosomes’ (Smc) complex Smc5/6 as being targeted by hepatitis B virus X protein for ligase-mediated degradation [27]. We hypothesized that a similar approach would serve to identify TRIM25 substrates, which will be immunoprecipitated more robustly with a TRIM25 E2 binding mutant than with TRIM25-WT, as the former is unable to mediate transfer of ubiquitin from E2 to substrates.

**Fig 1:**
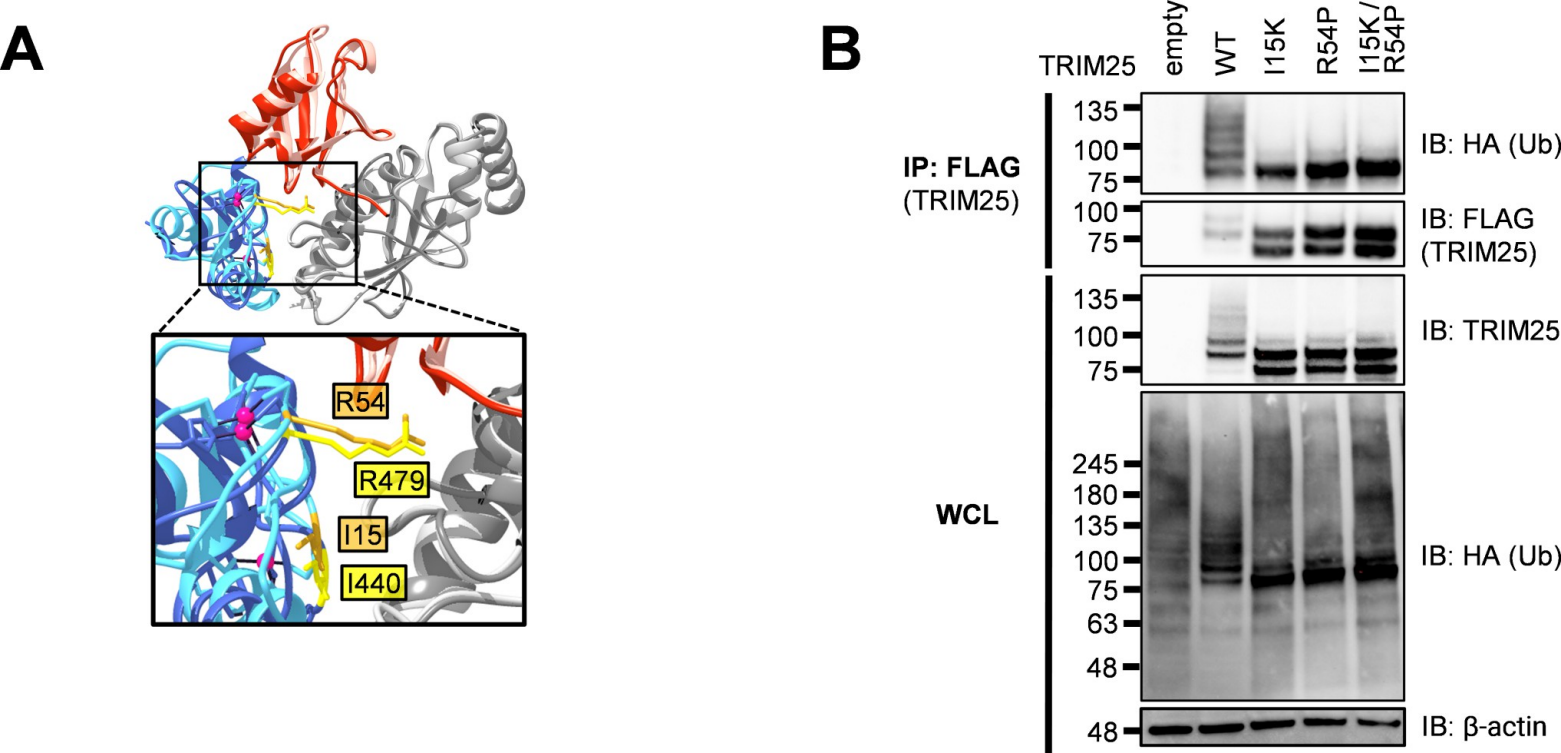
Individual TRIM25 RING residues required for TRIM25 autoubiquitination. (A) Alignment of the RING E3 ligases MDM2 (dark blue) and TRIM25 (light blue) in complex with ubiquitin (red) and the E2 UbcH5 (gray), performed using UCSF Chimera [30]. Highlighted in gold (TRIM25) and yellow (MDM2) are homologous residues. PDB: 5MNJ (MDM2), 5EYA (TRIM25). (B) Western blot of 293T cells transfected with FLAG-TRIM25 mutants and HA-ubiquitin (Ub). Lysates were subjected to FLAG IP. Data representative of three independent experiments.

Residues important for the RING-E2 interaction and thus necessary for ligase activity have already been identified in the RING E3 ligase MDM2 [28]. We aligned the structure of the TRIM25 RING domain complexed to E2-ubiquitin (Ub) to the analogous MDM2-E2-Ub structure and identified two conserved critical E2 interaction residues in TRIM25 RING, I15 and R54 (Fig 1A). To assess loss of ligase activity, we transfected HA-tagged Ub and FLAG-tagged TRIM25 into 293T cells and immunoprecipitated TRIM25 in denaturing conditions. We then blotted for HA-Ub, wherein polyubiquitination manifests as a ladder of bands. These TRIM25 E2 binding mutants (I15K and R54P), are deficient in auto-polyubiquitination, suggesting successful crippling of ligase activity (Fig 1B). Individual E2 binding mutants retain a mono-Ub band (Fig 1B), so we generated the double mutant I15K/R54P, which did not display further reduction in ligase activity (Fig 1B). Therefore, we selected the R54P mutant for future co-IP/MS studies since this mutation has previously been shown to reduce TRIM25 catalytic activity and polyubiquitin chain formation [29].

### Substrate trapping approach enriches for novel TRIM25 interactors

**Fig 2:**
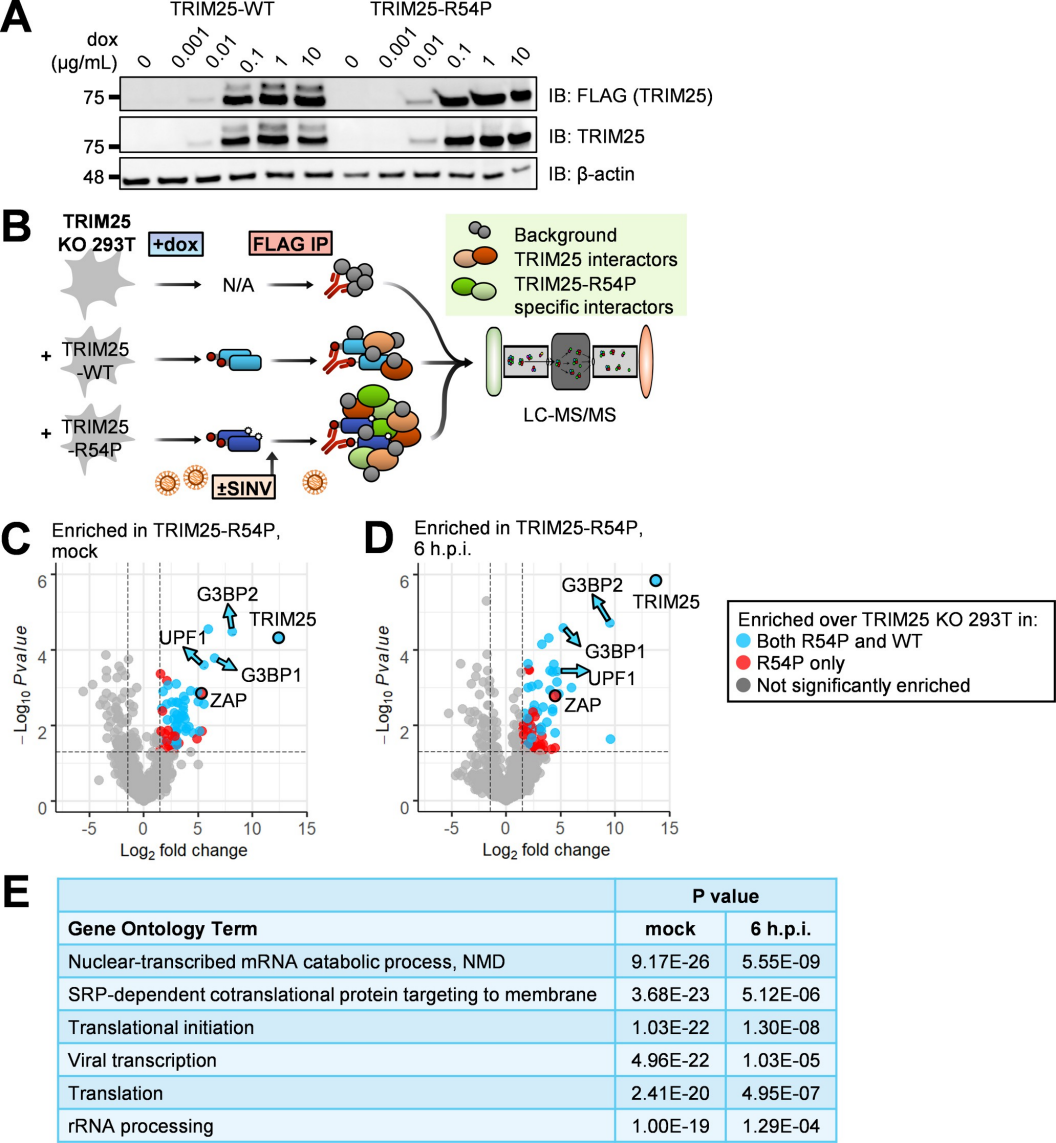
TRIM25 co-IP/MS identifies TRIM25 interactors. (A) Western blot of TRIM25-WT and -R54P doxycycline (dox)-inducible 293T cell lines in the presence of increasing amount of dox (0, 0.001, 0.01, 0.1, 1, and 10 μg/mL). Data are representative of two independent experiments. (B) Schematic of co-IP/MS experiment to identify TRIM25 interactors. (C-D) Volcano plots of proteins significantly enriched over TRIM25 KO background in TRIM25-R54P co-IP/MS in the (C) absence or (D) presence of viral infection. Data representative of two independent experiments. Blue dots represent proteins that were also significantly enriched in TRIM25-WT co-IP and red dots represent proteins that were only enriched in TRIM25-R54P co-IP. Proteins were counted as enriched when log2FC>1.5 and -log10Pvalue>1.3 (Pvalue<0.05). The R package EnhancedVolcano [32] was used to generate volcano plots. (E) Gene ontology terms significantly enriched in all unique TRIM25-WT and TRIM25-R54P interactors. Analysis performed for GO terms in biological processes using DAVID [33,34].

Next, we asked what proteins are modified by TRIM25, as identification of these substrates will elucidate how ubiquitination facilitates TRIM25-mediated cellular and antiviral activities. We first used CRISPR-Cas9 to generate a TRIM25 KO 293T cell line (S1 Fig). We then stably integrated doxycycline (dox) inducible FLAG-tagged TRIM25 wild-type (WT) and mutant R54P using the ePiggyBac (ePB) transposon system [31], where both TRIM25-WT and TRIM25-R54P are similarly induced in a dose-dependent manner (Fig 2A). TRIM25 protein levels are comparable upon detection using a FLAG or TRIM25-specific antibody (Fig 2A).

To capture TRIM25 substrates, we performed two independent co-IP/MS experiments using our reconstituted TRIM25 KO 293T cell lines (Fig 2B). We induced TRIM25-WT or -R54P expression in the presence or absence of the prototype alphavirus Sindbis virus (SINV), performed a FLAG IP to enrich for TRIM25, and analyzed the resultant protein mixture using MS. TRIM25 KO 293T cells with dox added were used as a control, since previous work by our lab found that dox treatment nonspecifically affects viral replication in other systems [35]. We found that this “substrate trapping” approach enriches for interactors specific to TRIM25-R54P under both mock and infected conditions (Fig 2C and 2D, red circles). These TRIM25-R54P-specific interactors tend to have lower fold change in abundance over background than interactors common to both TRIM25-WT and TRIM25-R54P (Fig 2C and 2D, blue circles), suggesting that the TRIM25-R54P co-IP/MS captures weaker interactions not identified with TRIM25-WT. After filtering for interactors enriched in both independent experiments, we found that TRIM25-R54P enriches for 14 unique interactors under mock conditions (Table 1) and that almost all TRIM25-WT interactors (25 of 30) are also present as TRIM25-R54P interactors (Table 2), indicating that TRIM25-R54P is otherwise functionally similar to TRIM25-WT. During viral infection, TRIM25-R54P enriches for all TRIM25-WT interactors in addition to 16 unique interactors (Tables 3 and 4), suggesting an effective “substrate trap.” Interestingly, we found that the number of TRIM25 interactors drastically decreases during viral infection for both TRIM25-WT (29 to 7 interactors; Tables 2 and 4) and TRIM25-R54P (38 to 23 interactors; Tables 1 and 3). We used DAVID bioinformatics resources [33,34] to find that TRIM25 interactors are highly enriched in GO terms involved in translation, RNA metabolism, and viral transcription (Fig 2E). This is in line with our hypothesis that TRIM25 substrates mediate diverse cellular and viral processes as a consequence of ubiquitination.

**Table 1:** TRIM25-R54P interactors in the absence of virus. Interactors pulled down in both independent experiments shown here; proteins also enriched in TRIM25-WT co-IP are italicized and bolded. Fold change = FC. In EXP #2, the “i” prefacing log2FC and Pvalue refers to how missing data values were imputed.

Protein	EXP #1 log2FC	EXP #2 ilog2FC	EXP #1 -log10Pvalue	EXP #2 -log10iPvalue
***TRIM25***	***12*.*39***	***7*.*51***	***4*.*29***	***2*.*12***
G3BP2	8.11	5.55	4.48	1.50
***G3BP1***	***6*.*50***	***4*.*03***	***3*.*78***	***1*.*73***
***PABPC1***	***5*.*93***	***5*.*10***	***4*.*55***	***3*.*28***
***UPF1***	***5*.*50***	***4*.*79***	***3*.*60***	***1*.*44***
RPL27	5.34	3.69	1.84	1.55
ZC3HAV1	5.32	5.31	2.83	1.42
***ZCCHC3***	***5*.*12***	***3*.*75***	***1*.*85***	***1*.*78***
RPL36	4.98	3.48	2.88	1.79
MOV10	4.91	4.49	1.64	1.80
***RPLP2***	***4*.*13***	***5*.*86***	***1*.*96***	***1*.*55***
***SSB***	***3*.*89***	***4*.*48***	***1*.*91***	***1*.*71***
***RPS3A***	***3*.*79***	***2*.*69***	***2*.*16***	***1*.*89***
***MRPL11***	***3*.*77***	***5*.*47***	***2*.*66***	***1*.*40***
RPL3	3.66	2.15	2.24	1.79
***RPS12***	***3*.*66***	***3*.*49***	***2*.*76***	***1*.*89***
***NME1***	***3*.*61***	***4*.*79***	***2*.*39***	***1*.*79***
***IGF2BP3***	***3*.*56***	***3*.*28***	***1*.*98***	***1*.*50***
***RPS8***	***3*.*26***	***3*.*27***	***2*.*19***	***1*.*75***
DNAJA1	3.17	3.92	2.61	1.91
RPL21	3.06	2.39	1.48	1.47
***DDX21***	***3*.*05***	***1*.*89***	***1*.*59***	***1*.*73***
***RPL7A***	***3*.*04***	***2*.*52***	***2*.*05***	***1*.*73***
***DDX50***	***3*.*03***	***3*.*66***	***1*.*48***	***1*.*53***
***RPL14***	***3*.*01***	***2*.*79***	***1*.*83***	***1*.*79***
***HSPA9***	***2*.*98***	***2*.*54***	***3*.*10***	***1*.*73***
***RPL8***	***2*.*95***	***2*.*64***	***2*.*62***	***1*.*50***
***RPL4***	***2*.*95***	***1*.*93***	***1*.*80***	***1*.*50***
***RPL30***	***2*.*92***	***4*.*99***	***1*.*86***	***1*.*81***
RPL6	2.88	1.95	1.97	1.50
RPL19	2.77	5.27	1.34	1.36
CXorf56	2.62	2.59	1.71	1.34
***MRPS25***	***2*.*56***	***3*.*34***	***2*.*61***	***1*.*54***
POLDIP2	2.24	2.45	3.07	1.64
***IGF2BP2***	***2*.*20***	***3*.*23***	***2*.*15***	***1*.*46***
***HSPA5***	***2*.*10***	***2*.*61***	***2*.*56***	***1*.*79***
NCL	1.95	2.58	1.78	2.63
***HSPA8***	***1*.*64***	***2*.*01***	***2*.*45***	***1*.*79***
RTRAF	1.60	1.76	1.84	1.31

**Table 2:** TRIM25-WT interactors in the absence of virus. Interactors pulled down in both independent experiments shown here; proteins also enriched in TRIM25-R54P co-IP in both independent experiments are italicized and bolded. Fold change = FC. In EXP #2, the “i” prefacing log2FC and Pvalue refers to how missing data values were imputed.

Protein	EXP #1 log2FC	EXP #2 ilog2FC	EXP #1 -log10Pvalue	EXP #2 -log10iPvalue
***TRIM25***	***12*.*35***	***7*.*21***	***4*.*29***	***2*.*36***
***NME1***	***5*.*89***	***5*.*69***	***3*.*19***	***2*.*11***
***PABPC1***	***5*.*18***	***4*.*36***	***4*.*32***	***2*.*65***
***UPF1***	***4*.*15***	***4*.*99***	***3*.*13***	***1*.*56***
***G3BP1***	***3*.*84***	***4*.*38***	***2*.*89***	***2*.*08***
***SSB***	***3*.*67***	***5*.*28***	***1*.*82***	***1*.*67***
***RPS12***	***3*.*52***	***3*.*02***	***2*.*70***	***2*.*24***
***DDX21***	***3*.*52***	***1*.*60***	***1*.*79***	***2*.*07***
***ZCCHC3***	***3*.*41***	***2*.*47***	***1*.*50***	***1*.*50***
***MRPL11***	***3*.*11***	***4*.*50***	***2*.*36***	***1*.*83***
***MRPS25***	***3*.*11***	***5*.*37***	***2*.*93***	***1*.*81***
***RPS3A***	***3*.*02***	***1*.*92***	***1*.*82***	***2*.*11***
***RPL14***	***2*.*91***	***2*.*56***	***1*.*78***	***1*.*71***
***RPS8***	***2*.*86***	***3*.*07***	***1*.*99***	***2*.*11***
RPL23A	2.83	2.50	1.42	2.29
LARP1	2.82	2.72	1.75	1.65
***HSPA9***	***2*.*78***	***2*.*55***	***2*.*98***	***1*.*98***
***DDX50***	***2*.*78***	***5*.*52***	***1*.*38***	***1*.*87***
***RPLP2***	***2*.*71***	***5*.*81***	***1*.*37***	***1*.*36***
***RPL7A***	***2*.*68***	***2*.*05***	***1*.*87***	***2*.*11***
***RPL8***	***2*.*67***	***1*.*91***	***2*.*46***	***1*.*46***
***IGF2BP3***	***2*.*67***	***3*.*65***	***1*.*56***	***1*.*66***
***HSPA5***	***2*.*36***	***2*.*77***	***2*.*75***	***2*.*05***
FMR1	2.16	2.14	1.38	1.56
***RPL4***	***2*.*09***	***1*.*96***	***1*.*33***	***2*.*08***
***RPL30***	***1*.*98***	***4*.*99***	***1*.*33***	***2*.*66***
RPL32	1.91	2.10	1.44	2.05
***IGF2BP2***	***1*.*73***	***2*.*16***	***1*.*80***	***1*.*45***
***HSPA8***	***1*.*66***	***2*.*26***	***2*.*46***	***1*.*65***
FXR1	1.52	4.86	2.66	1.59

**Table 3:** TRIM25-R54P interactors during viral infection. Interactors pulled down in both independent experiments shown here; proteins also enriched in TRIM25-WT co-IP in both independent experiments are italicized and bolded. Fold change = FC. In EXP #2, the “i” prefacing log2FC and Pvalue refers to how missing data values were imputed.

Protein	EXP #1 log2FC	EXP #2 ilog2FC	EXP #1 -log10Pvalue	EXP #2 -log10iPvalue
***TRIM25***	***13*.*82***	***6*.*87***	***5*.*83***	***1*.*28***
G3BP2	9.50	7.66	4.71	1.40
G3BP1	5.25	2.51	4.58	1.92
NME1	4.81	3.43	2.84	1.40
***H1FX***	***4*.*65***	***4*.*49***	***3*.*14***	***1*.*46***
***UPF1***	***4*.*64***	***5*.*24***	***3*.*44***	***1*.*94***
ZC3HAV1	4.47	2.77	2.77	1.42
RPL8	4.26	2.98	2.36	1.80
PABPC4	4.25	2.67	3.42	1.42
PABPC1	4.24	3.54	2.44	1.44
***HP1BP3***	***4*.*00***	***4*.*09***	***3*.*44***	***1*.*50***
***LARP1***	***3*.*93***	***4*.*59***	***4*.*32***	***1*.*61***
***DDX50***	***3*.*74***	***4*.*14***	***2*.*08***	***1*.*47***
RPL21	3.40	3.45	1.50	1.44
RPL29	3.38	2.53	2.48	2.06
***HSPA9***	***3*.*28***	***2*.*04***	***4*.*15***	***2*.*05***
***ZCCHC3***	***3*.*23***	***4*.*49***	***1*.*95***	***1*.*54***
RPS3A	3.18	1.53	1.36	1.38
GLYR1	3.01	2.59	1.43	1.59
YBX1	2.61	1.73	1.44	1.44
GNL3L	2.47	5.28	2.35	1.58
RPL19	2.44	2.90	2.39	1.75
MRPS26	1.98	1.90	1.49	1.31
MRPS9	1.58	2.56	2.02	1.38

**Table 4:** TRIM25-WT interactors during viral infection. Interactors pulled down in both independent experiments shown here; proteins also enriched in TRIM25-R54P co-IP in both independent experiments are italicized and bolded. Fold change = FC. In EXP #2, the “i” prefacing log2FC and Pvalue refers to how missing data values were imputed.

Protein	EXP #1 log2FC	EXP #2 ilog2FC	EXP #1 -log10Pvalue	EXP #2 -log10iPvalue
***TRIM25***	***13*.*19***	***6*.*43***	***5*.*75***	***1*.*15***
***H1FX***	***3*.*95***	***3*.*23***	***2*.*87***	***1*.*74***
***UPF1***	***3*.*86***	***5*.*51***	***3*.*13***	***1*.*62***
***HP1BP3***	***2*.*75***	***3*.*84***	***2*.*82***	***1*.*75***
***ZCCHC3***	***2*.*65***	***4*.*90***	***1*.*78***	***1*.*41***
***HSPA9***	***2*.*63***	***2*.*35***	***3*.*77***	***1*.*41***
***LARP1***	***2*.*62***	***4*.*61***	***3*.*63***	***1*.*42***
***DDX50***	***2*.*44***	***5*.*21***	***1*.*47***	***1*.*46***

### TRIM25 interacts with G3BP1 and 2 through a conserved binding motif and modifies them with predominantly K63 polyubiquitin chains

Among the most enriched TRIM25-R54P interactors in the presence and/or absence of SINV infection (Tables 1 and 3), we identified the core stress granule proteins G3BP1 and 2, RNA helicase UPF1 (Fig 2C and 2D, blue arrows), metastatic suppressor and nucleoside kinase NME1, and poly(A) binding protein PABPC4 as high priority candidates given our interest in RNA metabolic and translation processes (G3BP1 and 2, UPF1, PABPC4) and TRIM25’s role in regulating carcinogenesis (NME1). Next, we asked whether any of these are TRIM25 ubiquitination substrates.

Both G3BP1 and G3BP2, hereafter collectively referred to as G3BP, associate very strongly with TRIM25 in the co-IP/MS (Tables 1–4; G3BP1, log2FoldChange 2.5–6.5; G3BP2, log2FoldChange 5.5–9.5). G3BP normally function in stress granule (SG) assembly, interacting with RNA and other cellular proteins to induce SG formation [36,37]. Interestingly, the Old World alphaviruses exploit G3BP to promote their own replication [38–41]. These viruses utilize their non-structural protein 3 (nsP3) to recruit G3BP into viral replication complexes, which disrupts antiviral SG formation [42], clusters viral replication complexes [41], and recruits translation initiation machinery [43]. By doing so, alphaviruses enhance viral replication at the cost of endogenous G3BP function.

**Fig 3:**
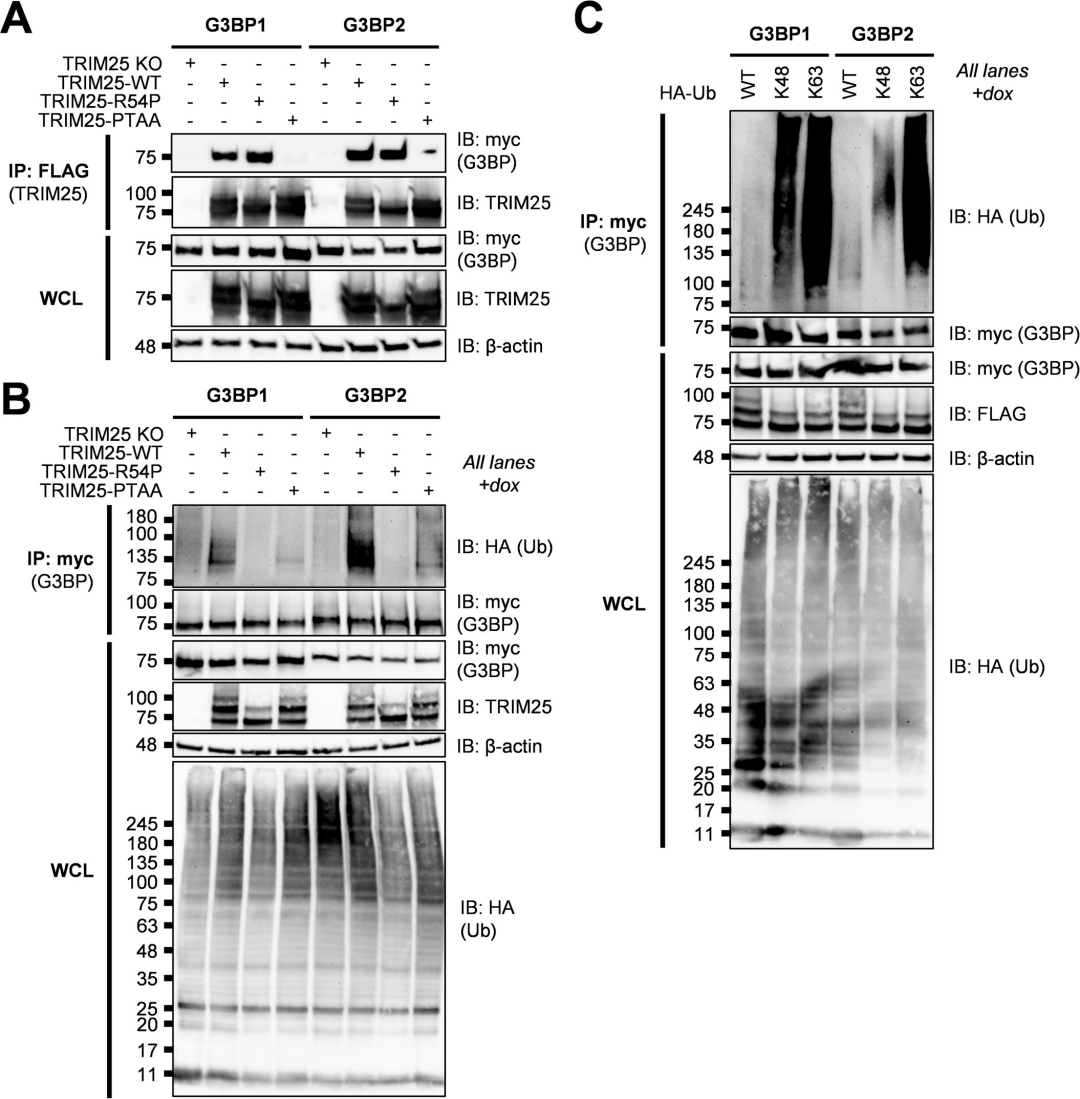
TRIM25 interacts with and polyubiquitinates G3BP. (A) Western blot of TRIM25 KO 293T cells transfected with myc-G3BP1/2 and FLAG-TRIM25-WT, -R54P, or -PTAA. Lysates were subjected to FLAG IP. Data are representative of three independent experiments. (B) Western blot of TRIM25 KO and TRIM25-WT, -R54P, or -PTAA inducible cells transfected with myc-G3BP1/2 and HA-Ub-WT in the presence of 1 μg/mL dox. (C) Western blot of TRIM25-WT inducible cells transfected with myc-G3BP1/2 and HA-Ub-WT, -K48, or -K63 in the presence of 1 μg/mL dox. (B-C) Lysates were subjected to myc IP. Data are representative of three independent experiments.

Previous work identified an FGDF peptide motif in alphavirus nsP3 which binds with high affinity to G3BP [42,44]. More recent work characterizing viral-host interaction motifs has uncovered a conserved G3BP-binding motif, ΦxFG (where Φ is a hydrophobic residue) [45]. This G3BP interaction motif is present in both viral and host proteins, such as the cellular SG protein and known G3BP interactor USP10, and is remarkably similar to the alphavirus nsP3-G3BP interaction motif, FGDF, but likely binds with lower affinity [45]. Moreover, TRIM25 was identified as a G3BP1 interaction partner [45]. Mutating the latter two amino acids in the TRIM25-specific motif (404-PTFG-407), to alanine (404-PTAA-407) was sufficient to abolish TRIM25-G3BP1 interaction [45]. Meanwhile, TRIM25 and G3BP2 have previously been shown to interact in the context of prostate cancer [46]. To examine whether this motif is also necessary for TRIM25-G3BP2 interaction, we co-transfected myc-tagged G3BP into TRIM25 KO 293T cells along with FLAG-tagged TRIM25-WT, -R54P, or -PTAA, and performed a FLAG IP to pull down TRIM25. While both TRIM25-WT and -R54P robustly associate with both G3BP1 and 2, TRIM25-PTAA does not associate with either G3BP1 or 2 (Fig 3A), validating our co-IP/MS identification of G3BP as TRIM25 interactors.

We then used the ePB transposon system to reconstitute TRIM25 KO 293T cells with dox inducible TRIM25-PTAA. To establish that TRIM25 ubiquitinates G3BP and that the TRIM25-G3BP interaction is necessary for ubiquitination, we co-transfected myc-tagged G3BP with HA-Ub into TRIM25-WT, -R54P, and -PTAA inducible cell lines. After inducing TRIM25 expression, we performed a myc IP and probed for the presence of ubiquitinated G3BP. We found that both G3BP1 and 2 are robustly polyubiquitinated only in the presence of TRIM25-WT (Fig 3B), again validating our co-IP/MS identification of G3BP as TRIM25 substrates. No ubiquitination is detected in the presence of ligase-deficient TRIM25-R54P, whereas ubiquitination is dramatically diminished in the presence of G3BP-interaction deficient TRIM25-PTAA (Fig 3B). Interestingly, TRIM25 appears to more robustly ubiquitinate G3BP2 as compared to G3BP1 (Fig 3B). Given the TRIM25-mediated polyubiquitination of G3BP1 and 2, we then characterized G3BP ubiquitination linkage type. To do so, we transfected our TRIM25-WT inducible cell line with myc-G3BP1 or -2 and different forms of HA-Ub: -WT, -K48, and -K63. Ub-K48 and -K63 have all lysines mutated to arginine except K48 and K63, respectively, such that only K48 or K63 polyubiquitin chains are able to be formed [47]. We found that both G3BP1 and 2 are most robustly ubiquitinated in the presence of Ub-K63, suggesting that TRIM25 primarily mediates K63-linked ubiquitination of both proteins (Fig 3C). Interestingly, while both G3BP1 and 2 exhibit a lower level of ubiquitination in the presence of Ub-K48, G3BP1 possesses more K48-linked polyubiquitin chains as compared to G3BP2 (Fig 3C), indicating that TRIM25 is able to distinguish between and differentially ubiquitinate these related proteins.

We then asked whether TRIM25-G3BP interaction and G3BP ubiquitination are required for TRIM25 antiviral activity. We found that overexpression of TRIM25-PTAA suppresses SINV replication (S2A Fig) and translation (S2B Fig) similarly to TRIM25-WT, suggesting that loss of TRIM25-G3BP interaction or G3BP ubiquitination is not sufficient to restore SINV infection. It has been demonstrated that different alphaviruses display differing degrees of dependency on G3BP for their replication, wherein SINV is partially reliant and chikungunya virus (CHIKV) is completely reliant on G3BP [41,48]. We hypothesized that a more G3BP-reliant virus such as CHIKV might be more sensitive to any antiviral mechanisms that are dependent on G3BP. In such a system, TRIM25-PTAA, which is unable to interact with and efficiently ubiquitinate G3BP to potentially disrupt their pro-viral functions, may not be as antiviral as TRIM25-WT. Interestingly, inhibition of CHIKV infection is also dependent on TRIM25 with functional ligase activity as TRIM25-R54P restores virion production to similar levels as TRIM25 KO (S2C Fig). However, we found no significant difference between TRIM25-WT and TRIM25-PTAA in their ability to suppress virion production (S2C Fig). Overall, though we validated G3BP interaction with and ubiquitination by TRIM25, we did not find that the TRIM25-G3BP axis is sufficient for TRIM25 antiviral activity.

### TRIM25 interacts with and mono-ubiquitinates UPF1 at K592

Moreover, UPF1 associates very strongly with TRIM25 in the co-IP/MS (Tables 1–4, log2FoldChange 3.9–5.5), supporting a role for UPF1 as a novel TRIM25 interactor. UPF1 is best known for its central role in nonsense-mediated mRNA decay (NMD), where it is recruited to premature termination codons to catalyze the NMD pathway, inhibiting further translation and recruiting other RNA-degrading enzymes [49]. UPF1 has also been implicated in serving an antiviral role in the context of alphavirus infection [50]. The authors of this study found that depletion of NMD components, including UPF1, promotes viral replication; further investigation revealed that UPF1 likely destabilizes incoming viral RNA genomes [50].

**Fig 4:**
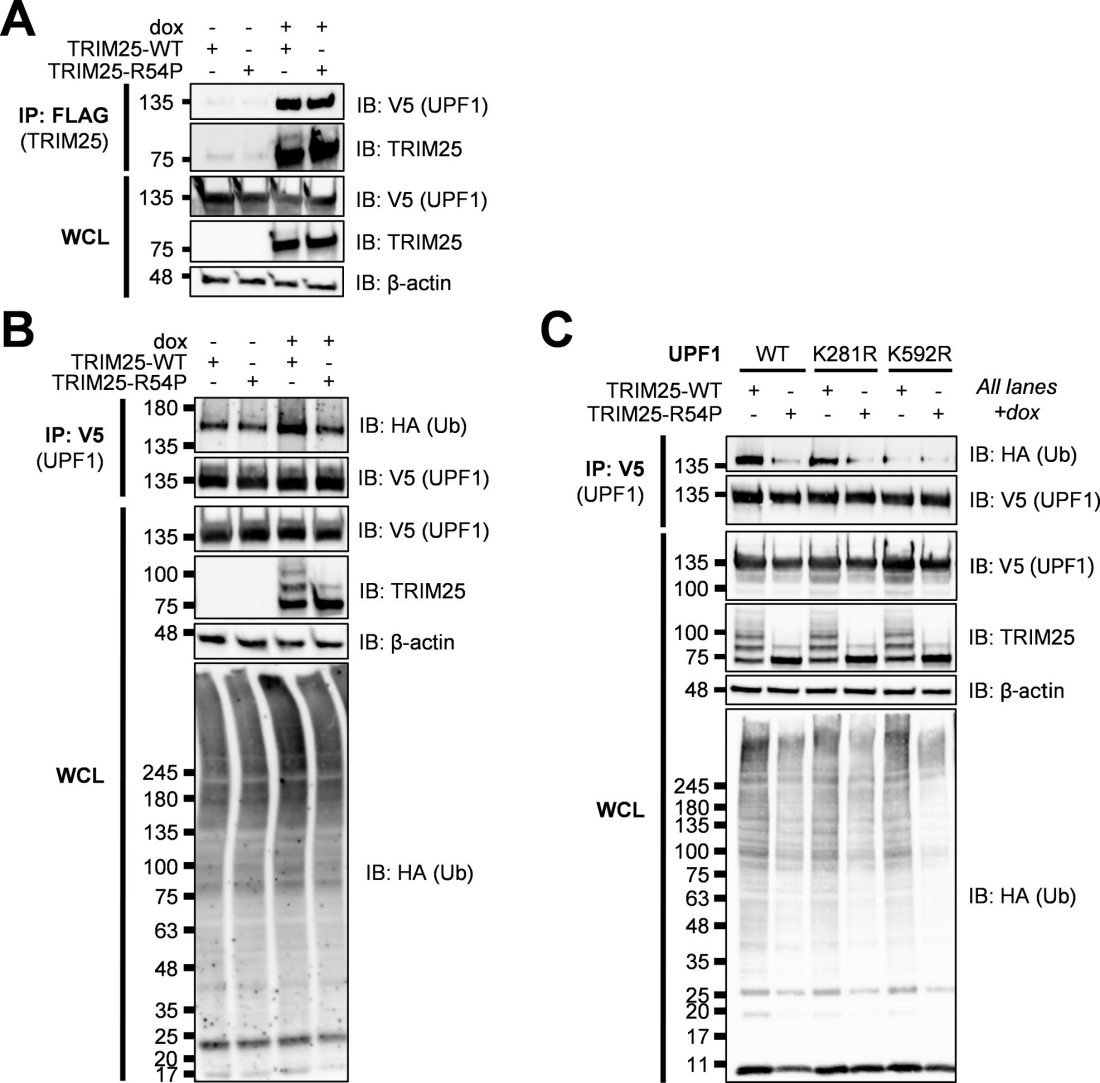
TRIM25 interacts with and mono-ubiquitinates UPF1. **(**A) Western blot of TRIM25 inducible cells transfected with V5-tagged UPF1 in the presence or absence of 1 μg/mL dox. Lysates were subjected to FLAG IP. Data are representative of three independent experiments. (B-C) Western blot of TRIM25 inducible cells transfected with (B) V5-UPF1 or (C) V5-UPF1 WT and mutants (K281R, K592R) and HA-Ub in the presence of 1 μg/mL dox. Lysates were subjected to V5 IP. Data are representative of two independent experiments for (B) and of three independent experiments for (C).

We first validated that TRIM25 interacts with UPF1. To do so, we transfected V5-tagged UPF1 into TRIM25 inducible cell lines, then induced for TRIM25-WT or -R54P expression with dox, and performed a FLAG IP to pull down TRIM25. We found that UPF1 is robustly detected only when TRIM25 is induced (Fig 4A), validating the TRIM25-UPF1 interaction identified in our co-IP/MS. To test the hypothesis that TRIM25 ubiquitinates UPF1, we co-transfected V5-tagged UPF1 with HA-Ub into our TRIM25 inducible cell lines and induced TRIM25 expression. We then performed a V5 IP and probed for the presence of ubiquitinated UPF1. We found that UPF1 is more robustly mono-ubiquitinated only in the presence of TRIM25-WT and not ligase-deficient TRIM25-R54P (~50% more by ImageJ quantification, Fig 4B), suggesting that TRIM25 mono-ubiquitinates UPF1. We then identified putative ubiquitination sites by selecting residues that are both identified in a previously published ubiquitinome [51] and predicted via UbPred to be ubiquitinated (Score > 0.70) [52], and mutated these sites to arginine (K281R, K592R). Whereas ubiquitination is unchanged in UPF1 K281R, the introduction of K592R abolishes UPF1 ubiquitination in the presence of TRIM25-WT (Fig 4C). Together, these results validate our co-IP/MS identification of UPF1 as a novel TRIM25 substrate.

Next, we asked whether UPF1 plays a role in TRIM25 antiviral activity. We tested several UPF1 siRNAs and selected the one with the most efficient knockdown (S2D Fig). We observed that UPF1 knockdown only has a significant effect on SINV replication when TRIM25 is absent, though trends toward an effect when TRIM25-WT is induced (S2E and S2F Fig). Together, these data suggest that UPF1 could be antiviral independent of TRIM25 and that it is not critical for the TRIM25 antiviral response.

### TRIM25 polyubiquitinates NME1 but only interacts with endogenous, not ectopically expressed NME1

Finally, we asked whether TRIM25-R54P specific interactors identified in our co-IP/MS were bona fide TRIM25 substrates. We identified NME1 as one of the most enriched TRIM25-R54P interactors in the presence of SINV infection (Table 3, log2FoldChange 3.4–4.8). NME1 is a nucleoside diphosphate kinase and a major synthesizer of non-ATP nucleoside triphosphates, perhaps best characterized in its role in inhibiting cell migration and proliferation of tumor cells via inhibition of MAPK signaling [53]. However, the role of NME1 in viral replication is not well studied [54].

**Fig 5:**
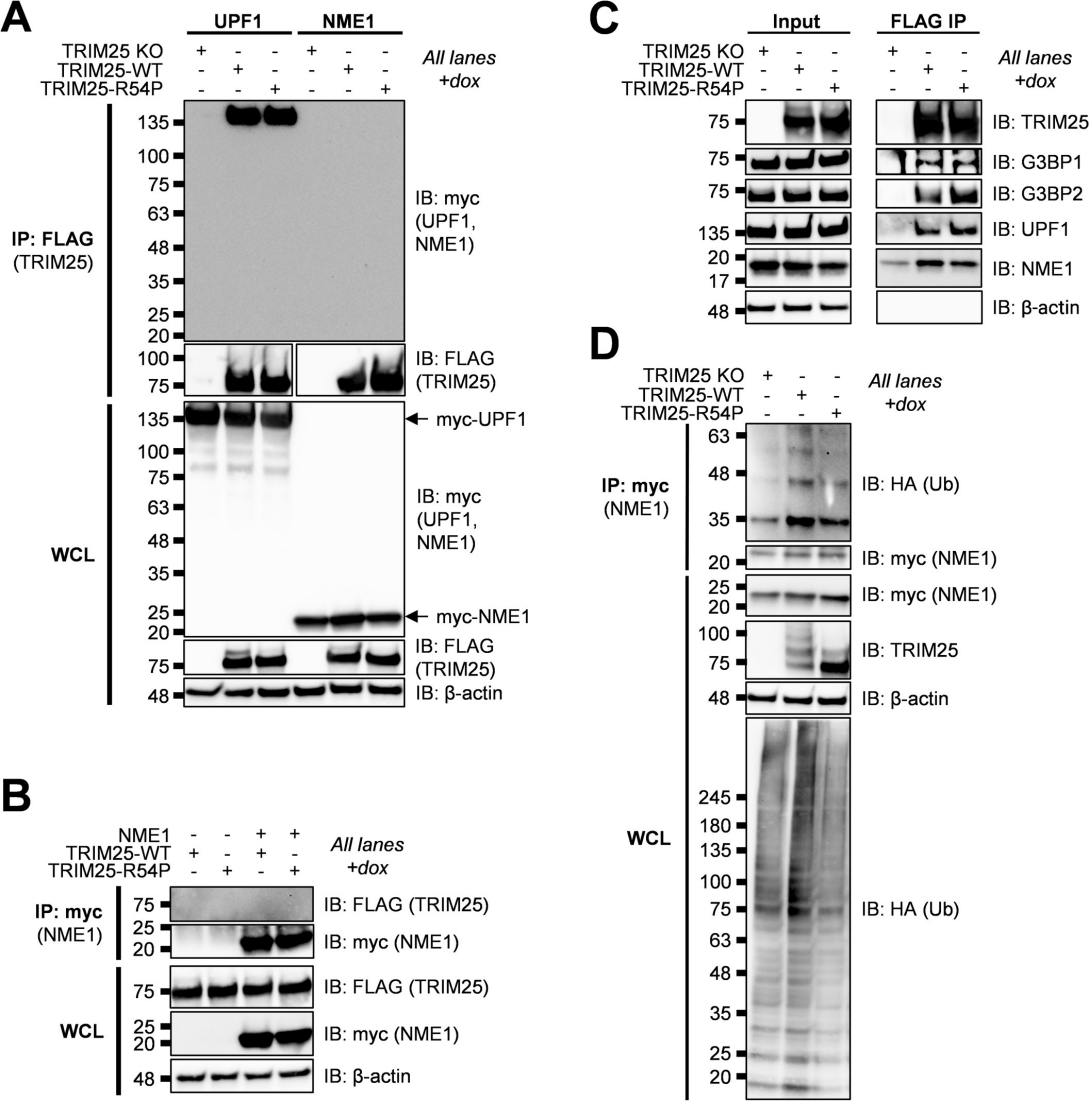
TRIM25 interacts with and polyubiquitinates NME1. (A) Western blot of TRIM25 KO and TRIM25 inducible cells transfected with myc-tagged UPF1 or NME1 in the presence of 1 μg/mL dox. Lysates were subjected to a FLAG IP. Data are representative of two independent experiments. (B) Western blot of TRIM25 inducible cells transfected with myc-NME1 in the presence or absence of 1 μg/mL dox. Lysates were subjected to a myc IP. Data are representative of two independent experiments. (C) Western blot of TRIM25 KO and TRIM25 inducible cells in the presence of 1 μg/mL dox. Lysates were subjected to a FLAG IP. Data are representative of two independent experiments. (D) Western blot of TRIM25 KO and TRIM25 inducible cells treated with 1 μg/mL dox and transfected with myc-NME1 and HA-Ub-WT. Lysates were subjected to myc IP. Data are representative of three independent experiments.

Given its well-characterized role as a metastatic suppressor, we decided to validate NME1 as a TRIM25 ubiquitination substrate. We first set out to validate TRIM25 interaction with NME1 as identified in our co-IP/MS (Tables 1–3). To do so, we transfected myc-tagged NME1 or UPF1 to serve as a positive control in our TRIM25 inducible lines, induced for TRIM25-WT or -R54P expression, and performed a FLAG IP to pull down TRIM25. We then probed for any associated UPF1 or NME1. While we saw robust association of UPF1 with both TRIM25-WT and -R54P in line with our previous results (Figs 4A and 5A; MW ~135 kDa), we did not identify NME1 (Fig 5A, MW 20–25 kDa). We also performed the reverse IP where we pulled down myc-tagged NME1, but were unable to find any TRIM25 interacting with NME1 (Fig 5B). We hypothesized that this lack of TRIM25-NME1 interaction could be due to functional differences between ectopically expressed myc-NME1 and endogenous NME1, given our successful validation of the other robust TRIM25 interactors from our co-IP/MS, G3BP and UPF1 (Figs 3 and 4). To test this hypothesis, we performed a FLAG IP using our TRIM25 inducible lines and probed for co-IP of endogenous NME1 along with endogenous G3BP and UPF1 as positive controls. In line with our co-IP/MS results, endogenous G3BP, UPF1, and NME1 enrich robustly with TRIM25 pulldown, despite a low level of non-specific binding of NME1 to the FLAG IP in TRIM25 KO 293T cells (Fig 5C).

To test whether TRIM25 ubiquitinates NME1, we transfected myc-tagged NME1 into TRIM25-WT and -R54P inducible cells, induced for TRIM25 expression, and performed a myc IP. We found that NME1 is more robustly polyubiquitinated in the presence of TRIM25-WT as compared to TRIM25-R54P, although we cannot yet rule out the possibility that TRIM25 might mono-ubiquitinate NME1 at multiple sites (Fig 5D).

### TRIM25 interacts with PABPC4 and predominantly modifies it with K63 polyubiquitin chains

We chose PABPC4 as a second example of TRIM25-R54P specific substrate, which was identified as a TRIM25-R54P interactor in the presence of SINV infection (Table 3). PABPC4 is a member of the poly(A) binding protein (PABP) family, which functions in translation initiation by binding the mRNA poly(A) tail, thus regulating mRNA translation and stability [55]. PABPs have been shown to localize to SGs and to inhibit recruitment of UPF1 to 3’UTRs [55,56]. Given their key roles in translation and mRNA metabolism, PABPs are frequently targeted and manipulated by viruses during infection [57]. Interestingly, PABPC4 was recently found to broadly inhibit coronavirus replication by recruiting an E3 ligase to ubiquitinate the viral nucleocapsid protein and target it for degradation [58].

**Fig 6:**
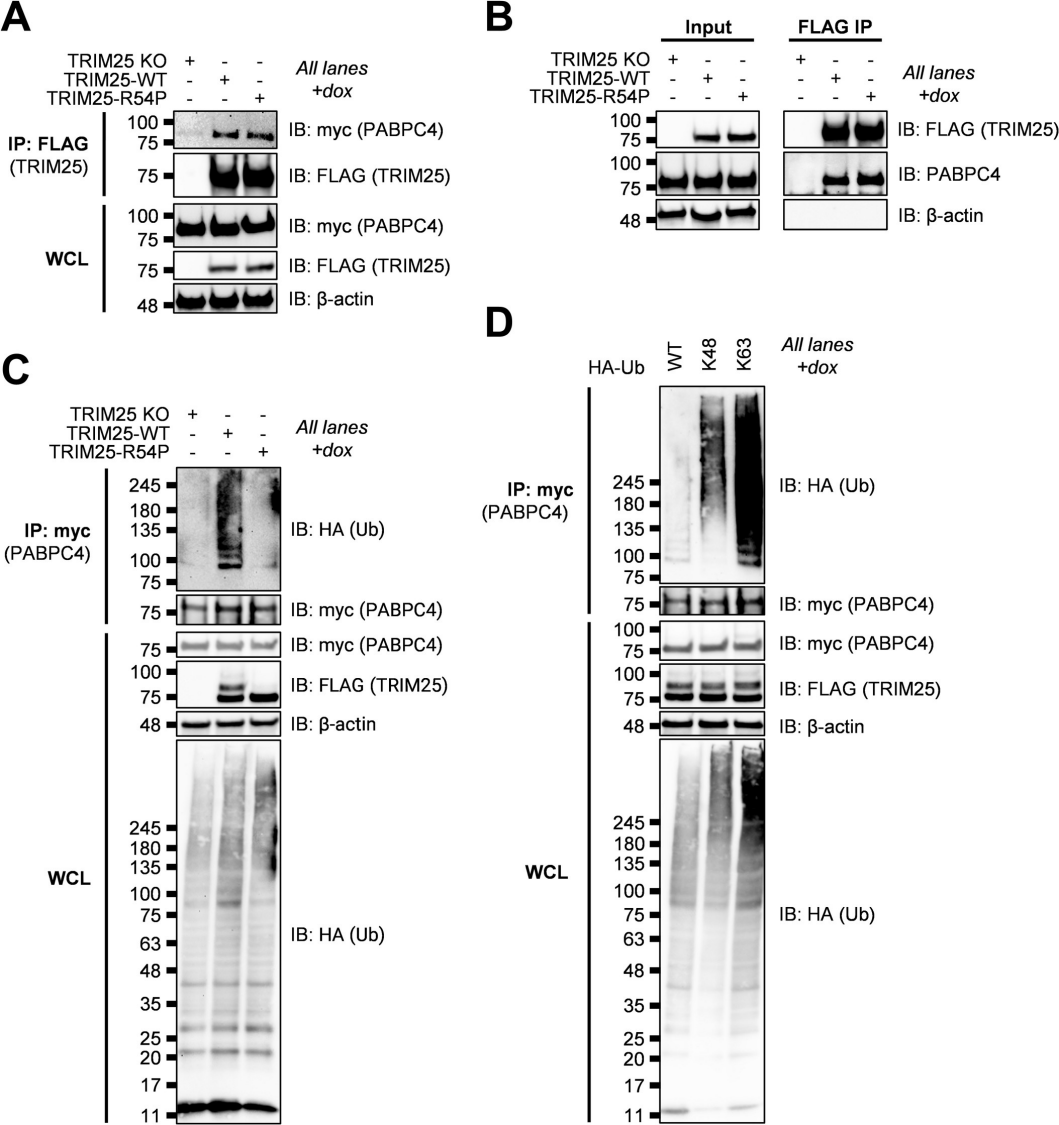
TRIM25 interacts with and polyubiquitinates PABPC4. (A) Western blot of TRIM25 KO and TRIM25 inducible cells transfected with myc-tagged PABPC4 in the presence of 1 μg/mL dox. Lysates were subjected to a FLAG IP. Data are representative of two independent experiments. (B) Western blot of TRIM25 KO and TRIM25 inducible cells in the presence of 1 μg/mL dox. Lysates were subjected to a FLAG IP. Data are representative of two independent experiments. (C) Western blot of TRIM25 KO and TRIM25 inducible cells treated with 1 μg/mL dox and transfected with myc-PABPC4 and HA-Ub-WT. Lysates were subjected to myc IP. Data are representative of two independent experiments. (D) Western blot of TRIM25-WT inducible cells treated with 1 μg/mL dox and transfected with myc-PABPC4 and HA-Ub-WT, -K48, or -K63. Lysates were subjected to myc IP. Data are representative of two independent experiments.

We first validated the TRIM25-PABPC4 interaction by transfecting myc-tagged PABPC4, induced for TRIM25-WT or -R54P expression, and performed a FLAG IP to pull down TRIM25. We then probed for any associated PABPC4. We found that PABPC4 is robustly detected when either TRIM25-WT or TRIM25-R54P is induced (Fig 6A), validating the TRIM25-PABPC4 interaction identified in our co-IP/MS. We also found that both TRIM25-WT and -R54P interact with endogenous PABPC4 (Fig 6B).

We then asked whether TRIM25 ubiquitinates PABPC4. We transfected myc-PABPC4 into TRIM25-WT and -R54P inducible cells, induced for TRIM25 expression, and performed a myc IP. We found that PABPC4 is more robustly polyubiquitinated in the presence of TRIM25-WT as compared to TRIM25-R54P (Fig 6C). Upon characterizing ubiquitination via transfection of Ub-K48 or -K63, we found that like G3BP, PABPC4 is most robustly ubiquitinated in the presence of Ub-K63, suggesting that TRIM25 primarily mediates K63-linked ubiquitination of PABPC4 (Fig 6D).

Taken together, these results suggest that TRIM25-R54P specific interactors identified in our co-IP/MS, such as NME1 and PABPC4, function as bona fide TRIM25 substrates, and that TRIM25 is able to utilize a range of ubiquitin linkages dependent on the substrate context.

### TRIM25 antiviral activity is dependent on its ligase activity

Given our identification of diverse host factors as TRIM25 substrates (Figs 3–6), many of which function in translational and RNA processes (Fig 2E) and several of which have known roles in alphavirus replication, we hypothesized that TRIM25 ligase activity is critical for orchestrating an antiviral response.

**Fig 7:**
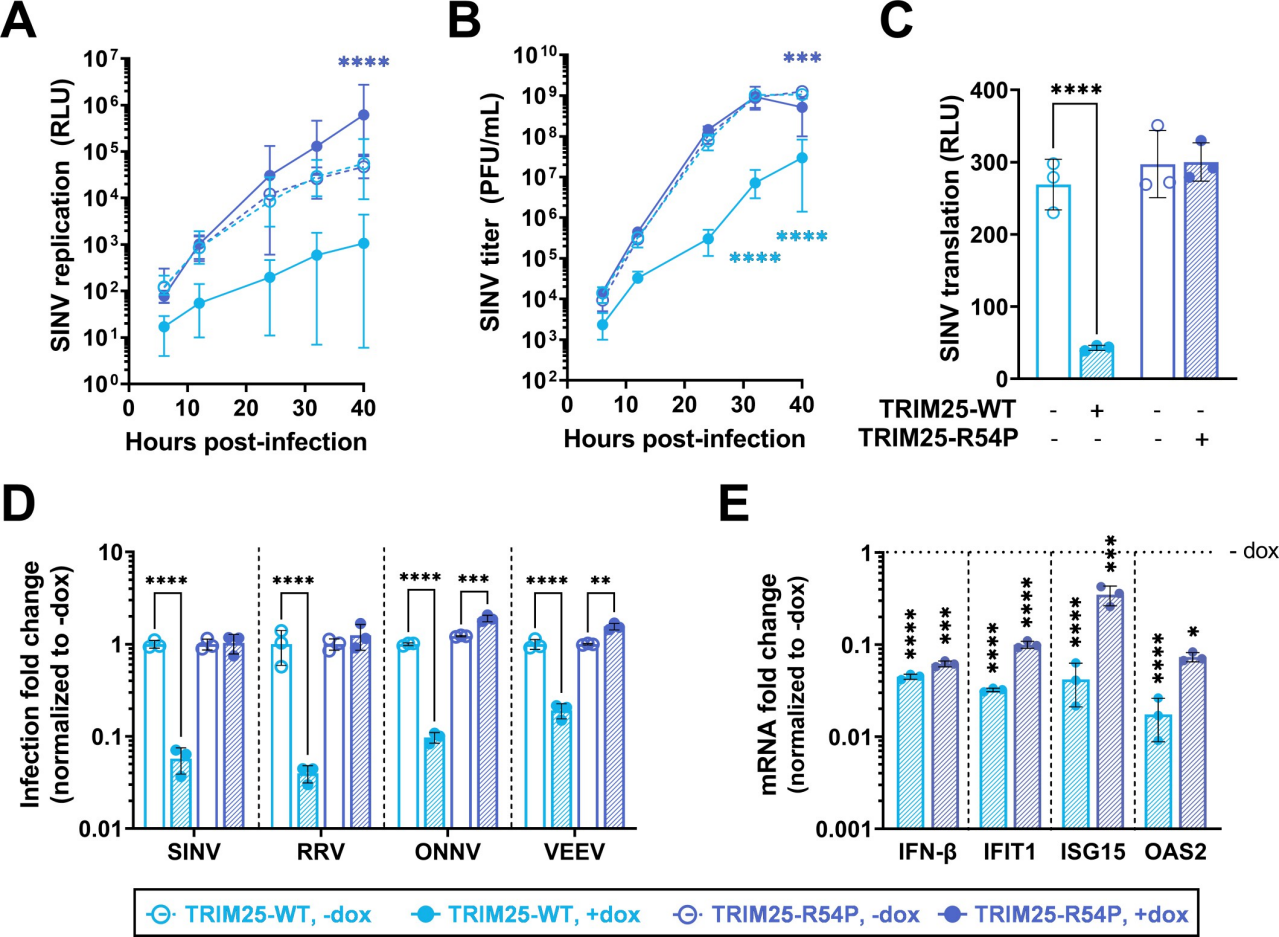
Point mutation in TRIM25 RING domain cripples TRIM25 antiviral activity. (A-C) Dox inducible TRIM25-WT or -R54P cells were induced for TRIM25-WT or -R54P expression at 1 μg/mL dox. Cells were infected with (A) SINV Toto1101/Luc at an MOI of 0.01 plaque forming unit (PFU)/cell, and lysed at 6, 12, 24, 32, and 40 hours post infection (h.p.i.); data combined from three independent experiments, error bars indicate range; or (B) Sindbis virus (SINV) Toto1101 at an MOI of 0.01 PFU/cell, harvesting supernatant at 6, 12, 24, 32, and 40 h.p.i. for plaque assays; data representative of two independent experiments, error bars indicate range; or (C) SINV Toto1101/Luc:ts6 at an MOI of 1 PFU/cell and lysed at 6 h.p.i. for measurement of luciferase activity; data representative of two independent experiments, error bars indicate standard deviation. (D) Percent infected cells (GFP+) at MOI of 0.01 PFU/cell (SINV 24 h.p.i.; Ross River virus (RRV) 24 h.p.i.; o’nyong-nyong virus (ONNV) 22 h.p.i.; Venezuelan equine encephalitis virus (VEEV) 10 h.p.i.) were normalized to that of the respective cell line without dox (set to one-fold). Asterisks indicate statistically significant differences, calculated using (A-B, D) Two-way ANOVA and Tukey’s multiple comparisons test: **, p<0.01; ***, p<0.001; ****, p<0.0001; (light blue compares WT +/- dox, dark blue compares R54P +/- dox) or (C) Two-way ANOVA and Šidák’s multiple comparisons test: ****, p<0.0001. Data for each virus (demarcated by dashed lines) was statistically analyzed independently. (E) TRIM25 inducible cells were treated with poly(I:C) in the presence or absence of dox, and RNA was harvested for RT-qPCR analysis. mRNA levels of IFN/ISGs in TRIM25-WT or R54P were normalized to that of the respective cell line without dox (set to one-fold, horizontal dotted line). Data representative of two independent experiments. mRNA fold change for each gene (demarcated by vertical dashed lines) was statistically analyzed independently. Asterisks indicate statistically significant differences as compared to the -dox condition (Two-way ANOVA and Šidák’s multiple comparisons test: *, p<0.05; ***, p<0.001; ****, p<0.0001).

We used TRIM25 inducible cell lines in the KO background (Fig 2A) to characterize the requirement of ligase activity in TRIM25-mediated viral inhibition. We found that TRIM25-WT, which retains ligase activity, represses SINV replication, whereas ligase mutant TRIM25-R54P does not (Fig 7A). Overexpression of TRIM25-WT (Fig 7A, solid light blue line) dramatically represses SINV replication by 7–15 fold at earlier timepoints (6–12 hours post infection (h.p.i.)) to 43–52 fold at later timepoints (24–40 h.p.i.) compared to TRIM25 KO 293T cell lines (Fig 7A, dotted lines). Interestingly, some replicates fail to initiate infection in the presence of TRIM25-WT, causing seemingly large variability in viral replication. In contrast, overexpression of ligase-deficient TRIM25-R54P (Fig 7A, solid dark blue line) restores SINV replication to levels even higher than the TRIM25 KO background (Fig 7A, dotted lines). Overexpressed TRIM25-R54P may act in a dominant negative manner by binding to and sequestering ZAP, preventing ZAP from interacting with its other co-factors. Similarly, we found that overexpression of TRIM25-WT robustly represses virion production by approximately 36–250 fold at 24–40 h.p.i., whereas overexpression of TRIM25-R54P restores virion production to comparable levels as the TRIM25 KO background (Fig 7B, compare solid light blue line to solid dark blue line).

We then investigated at which step TRIM25 may be acting to inhibit SINV infection. Previous work done by our lab showed that TRIM25 synergized with ZAP in blocking SINV translation [20]. We utilized a temperature-sensitive replication-deficient SINV luciferase reporter virus to characterize the requirement of ligase activity in TRIM25-mediated inhibition of viral translation, since luciferase activity in infected cell lysates represents translation of the incoming viral genome. Overexpressed TRIM25-WT inhibits viral translation by 6 fold at 6 h.p.i. (Fig 7C), supporting our hypothesis that TRIM25 blocks alphavirus replication by inhibiting translation of incoming viral genomes.

While we already examined TRIM25 antiviral activity against the Old World alphavirus CHIKV, wherein TRIM25-WT inhibits robustly and TRIM25-R54P fails to inhibit (S2C Fig), we then asked whether ligase-deficient TRIM25-R54P remains active against other alphaviruses. We tested other Old World (Ross River virus, RRV; o’nyong-nyong virus, ONNV) and New World (Venezuelan equine encephalitis virus, VEEV) alphaviruses. TRIM25-WT remains potently antiviral against all alphaviruses tested (Fig 7D, light blue shaded bar), while overexpression of TRIM25-R54P either has no effect on or restores viral replication to levels higher than the TRIM25 KO background (Fig 7D, dark blue shaded bar). Taken together, these data clearly demonstrate that TRIM25-dependent ubiquitination is required for inhibition of alphavirus replication, specifically through a block in viral translation.

### TRIM25-mediated viral inhibition is independent of changes in the type I IFN response

To exclude the complementary possibility that TRIM25 is exerting antiviral effects through affecting type I IFN or ISG production, we quantified the mRNA of IFN-β and the prominent ISGs IFIT1, ISG15, and OAS2 in the presence of poly(I:C), a dsRNA mimetic and stimulator of innate immune signaling. If TRIM25 antiviral activity is mediated through a strengthened IFN response, we would expect that both IFN and ISG production to increase when TRIM25-WT is induced and to be lower in the presence of TRIM25-R54P due to its defective antiviral activity. Poly(I:C) stimulation works well, inducing IFN-β robustly in the presence and absence of TRIM25 induction (S3 Fig). Surprisingly, we found that overexpression of either TRIM25-WT or TRIM25-R54P significantly suppresses production of IFN-β, IFIT1, ISG15, and OAS2 mRNA in the presence of poly(I:C) (Fig 7E). We also observed that induction of TRIM25-WT results in a more drastic suppression of the ISGs as compared to TRIM25-R54P (Fig 7E, compare light blue to dark blue shaded bar), leading to a higher type I IFN response in the TRIM25-R54P inducible cell line. Together, these data support our hypothesis that TRIM25 antiviral activity is not mediated through the IFN response.

### Identifying TRIM25-R54P specific interactors as critical for viral inhibition

**Fig 8:**
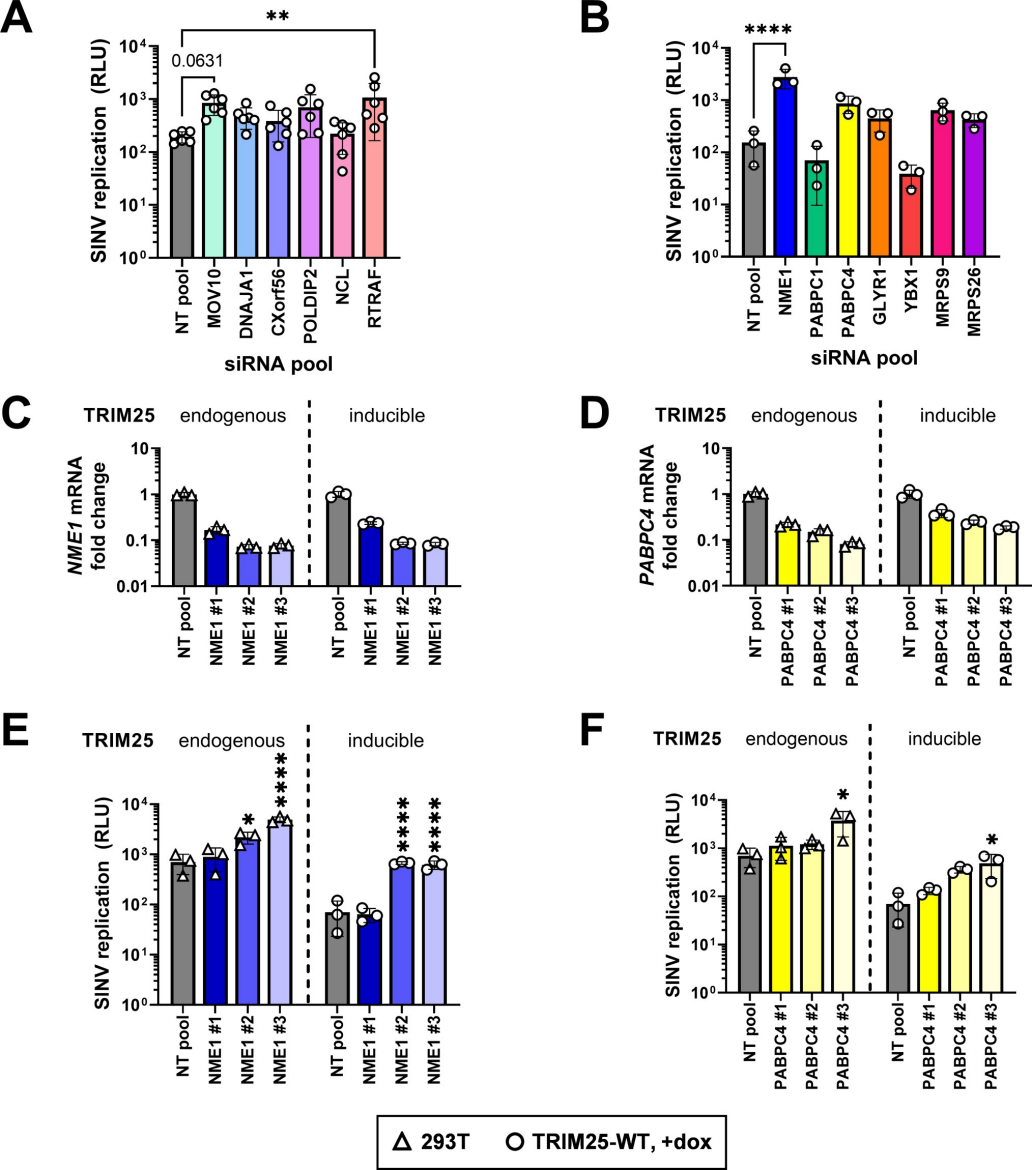
Knocking down TRIM25-R54P-specific interactors identifies essential substrates for TRIM25 antiviral activity. (A**-**B) TRIM25 inducible cells were transfected with pooled siRNAs for either (A) hits specific to TRIM25-R54P in the absence of viral infection or (B) hits specific to TRIM25-R54P in the presence of viral infection. Cells were induced for TRIM25-WT expression at 1 μg/mL dox, infected with Toto1101/Luc at an MOI of 0.01 PFU/cell, and lysed at 24 h.p.i. for measurement of luciferase activity. Asterisks indicate statistically significant differences as compared to the NT pool siRNA (One-way ANOVA, Dunnett’s multiple comparison test; **, p<0.01; ****, p<0.0001). Unlabeled comparisons are not significant. Data are either (A) pooled from or (B) representative of two independent experiments. (C-F) Parental 293T cells (TRIM25: endogenous) or TRIM25 inducible (TRIM25: inducible) cells were transfected with individual siRNAs for (C,E) *NME1* or (D,F) *PABPC4*, induced for TRIM25-WT expression at 1 μg/mL dox, and (C-D) had RNA extracted for RT-qPCR analysis or (E-F) infected with Toto1101/Luc at an MOI of 0.01 PFU/cell. Cells were lysed at 24 h.p.i. for measurement of luciferase activity. Asterisks indicate statistically significant differences as compared to the NT pool for each cell line. 293T and TRIM25-WT inducible cell lines were statistically analyzed independently from one another (One-way ANOVA, Dunnett’s multiple comparison test; *, p<0.05; ****, p<0.0001). Data are representative of two independent experiments for each cell line.

As we showed that the loss of antiviral activity of TRIM25-R54P does not correlate with the levels of IFN and ISG expression, suggesting a direct consequence of TRIM25-mediated ubiquitination of target proteins, we then decided to examine TRIM25-R54P interactors identified in our co-IP/MS that are not consistently present in the TRIM25-WT enrichment (non-bolded and non-italicized proteins; Tables 1 and 3). These candidate proteins likely exhibit weaker or more transient interactions with TRIM25 and are ubiquitinated by TRIM25. We hypothesized that if any of these interactors are critical for TRIM25 antiviral activity, loss of their expression would result in increased viral replication even in the presence of overexpressed TRIM25-WT. While we initially also assessed a subset of ribosomal proteins identified as TRIM25-R54P interactors, their knockdown results in high cytotoxicity and therefore are excluded from subsequent analyses. We validated most of the TRIM25-R54P interactors that are not present on the TRIM25-WT lists (Tables 1–4) in the absence (Table 1 and Figs 8A and S4A) or presence of viral infection (Table 3 and Figs 8B and S4B). While knockdown of multiple interactors trends towards restoring SINV replication, only loss of RTRAF (Table 1, log2FoldChange 1.6–1.8) and NME1 (Table 3, log2FoldChange 3.4–4.8) significantly restores SINV replication (Fig 8A and 8B). Moreover, knockdown of MOV10 (Table 1, log2FoldChange 4.5–4.9) approaches significant restoration of SINV replication (Fig 8A, p = 0.0631).

We decided to de-convolute the siRNA pools for both NME1 and PABPC4, given our verification of them as bona fide TRIM25 interactors and substrates (Figs 5 and 6). Moreover, loss of NME1 results in the most significant restoration of SINV replication (Fig 8B). We hypothesized that siRNAs that induced greater knockdown of *NME1* or *PABPC4* expression would also result in greater SINV replication. Therefore, we de-convoluted both *NME1* and *PABPC4* siRNA pools in both our inducible TRIM25-WT cell line and in the parental 293T cell line with endogenous TRIM25 and ZAP expression. There, we observed that both the degree of *NME1* and *PABPC4* mRNA expression (Fig 8C and 8D) significantly and negatively correlate with increase of viral replication (Fig 8E and 8F), supporting a role for both NME1 and PABPC4 in TRIM25-dependent alphavirus inhibition. This correlation is more robust in the presence of inducible TRIM25-WT (NME1: r = -0.83, p<0.001; PABPC4: r = -0.87, p<0.001) than in the presence of endogenous TRIM25 (NME1: r = -0.82, p<0.01; PABPC4: r = -0.74, p<0.01). Altogether, these results suggest that the antiviral activity of TRIM25 is mediated by multiple substrates. Though knockdown of most individual interactors on their own does not significantly restore SINV replication, the fact that several have demonstrated a phenotype implies that together they may have a larger impact on viral replication. Further studies need to be performed to determine their synergistic effects on viral infection and functional consequences of their ubiquitination by TRIM25.

## Discussion

Many TRIMs are involved in and ubiquitinate components of multiple cellular and antiviral processes [3–6]. In this study, we set out to identify TRIM25 substrates by generating a point mutation in the TRIM25 RING domain, R54P, which is predicted to abolish its interaction with E2 carrier enzymes and is sufficient to cripple TRIM25 ligase activity (Fig 1). We reported identification of TRIM25 substrates involved in nucleic acid metabolism and translation (Fig 2E), in line with its role in blocking viral translation [20]. We characterized the ubiquitination of the most enriched TRIM25 interactors, G3BP (Fig 3) and UPF1 (Fig 4), as well as two TRIM25-R54P specific interactors during infection, NME1 (Fig 5), and PABPC4 (Fig 6). These represent proteins with essential cellular functions, some of which with prior involvement in alphavirus infection [50,59]. We also used the TRIM25-R54P mutant to definitively show the critical role of ubiquitination in TRIM25 antiviral activity that is independent of IFN production and signaling (Fig 7). We then examined proteins that display a preference for association with TRIM25-R54P under mock and viral infection conditions, and found that several of these are necessary for TRIM25 antiviral activity (Fig 8), identifying them as potential TRIM25 substrates mediating viral inhibition. Our results suggest that targeting of any single substrate by TRIM25 is insufficient to mediate the entirety of its cellular and antiviral activities, illustrating the powerful, multi-faceted role of this ubiquitination network in diverse biological processes.

We propose that the success of this “substrate trapping” approach in identifying TRIM25 ubiquitination substrates hinges on preservation of protein structure. Previous reports that unearthed the importance of TRIM25 ligase activity in the ZAP antiviral response depend on either deleting the entire TRIM25 RING catalytic domain or disrupting formation of the zinc finger motif, potentially having an adverse effect on protein folding overall and potentially affecting other TRIM25 cellular functions or interactions [20,21]. The R54P point mutation we generated has been demonstrated to preserve protein structure and cognate interactions in other contexts [29], instilling greater credibility in our identification of novel TRIM25 substrates. Moreover, this mutation is predicted to abolish the E3 ligase-E2 conjugating enzyme interaction [29], preventing any downstream ubiquitination events and thus prolonging transient ligase-substrate interactions. The TRIM25-R54P specific hits may have weaker, more transient, or infection-specific interactions not easily detected by the conventional co-IP/MS approach. Other “substrate trapping” approaches depend on fusing a polyubiquitin binding domain to the ligase of interest [60], which may either disrupt native protein-protein interactions or result in false-positive identification of ubiquitinated proteins. Moreover, this type of approach would fail to identify substrates that are not polyubiquitinated, given that ligases can mono or multi-monoubiquitinate their substrates [61].

For the first time, we identified G3BP1/2, UPF1, NME1, and PABPC4 as bona fide TRIM25 substrates (Figs 3–6). Furthermore, we were able to characterize TRIM25 polyubiquitination of G3BP and PABPC4 as primarily utilizing K63 linkages (Figs 3C and 6D). This type of linkage is commonly used to build signaling scaffolds, as TRIM25 does to activate RIG-I [19], and could potentially play a role in either SG assembly or disassembly by recruiting SG components in the former or generating steric hindrance in the latter. Additionally, our validation of K592 as a mono-ubiquitination site on UPF1 (Fig 4C) overlaps with a predicted acetylation site on the same residue, and neighbors a predicted phosphorylation site at T595, potentially modulating these other post-translational modifications of UPF1 [62]. These residues lie within the AAA ATPase domain of UPF1, suggesting that ubiquitination of UPF1 by TRIM25 might affect its ATP hydrolysis, thus hindering UPF1 in its NMD target discrimination and efficient translation termination [63,64]. Interestingly enough, G3BP1 and UPF1 cooperate to mediate structure-mediated RNA decay [65]. It is entirely possible that TRIM25-mediated ubiquitination could affect this process by modulating their interaction with one another, though further experiments are required to explore this hypothesis.

Though UPF1 and G3BP have previously been implicated as antiviral and pro-viral factors in alphavirus replication, respectively [50,59], we did not find a role for either in the TRIM25-ZAP antiviral response. UPF1 may have an antiviral role independent of TRIM25, given that its knockdown only significantly rescued viral replication when TRIM25 was absent (S2D Fig). This hypothesis is supported by a previous report which found that UPF1 was involved in regulating half-life stability of viral RNA [50], which TRIM25 does not affect [20], On the other hand, G3BP is known to cluster replication complexes and recruit translation initiation machinery [59], which might be disrupted through G3BP ubiquitination by TRIM25, resulting in translational suppression. Alternatively, given that the G3BP interaction motifs in TRIM25 and nsP3 are similar [45,66], it is tempting to speculate that TRIM25 may compete with the viral nsP3 for G3BP interaction and recruitment, resulting in diminishment of G3BP pro-viral effects. However, the pro-viral roles of G3BP did not seem to be affected by TRIM25 ubiquitination or lack thereof, given that abolishing TRIM25-G3BP interaction through overexpression of the TRIM25-PTAA mutant did not rescue SINV replication and translation nor CHIKV virion production (S2A–S2C Fig). Nevertheless, we noted that the TRIM25-PTAA mutant still preserves some ubiquitination of G3BP despite completely abolishing the TRIM25-G3BP interaction (Fig 3A and 3B). Further studies are warranted to fully elucidate the role of G3BP ubiquitination in TRIM25 antiviral activity.

TRIM25-mediated ubiquitination of NME1 and PABPC4 may interfere with RNA metabolic processes by altering their stability or ability to bind RNA. Both of these proteins have previously been demonstrated to be ubiquitinated by other E3 ligases. Ubiquitination of NME1 by the E3 ligase SCF-FBXO24 targets it for degradation [67]. Seeing as TRIM25 is able to modify G3BP with both proteolytic K48- and non-proteolytic K63-polyubiquitin linkages, TRIM25 may also be targeting NME1 for degradation, thereby hindering nucleotide synthesis and general RNA metabolic processes. On the other hand, ubiquitination of PABPC4 by the E3 ligase MKRN decreases its affinity for binding mRNA poly(A) tails [68]. It is interesting to speculate that TRIM25-mediated polyubiquitination of PABPC4 could regulate PABPC4 binding to the poly(A) tail on viral RNAs, thus modulating the stability of the RNA and reducing its ability to form translation initiation complexes.

We also utilized the TRIM25-R54P mutant to define the requirement for ligase activity in TRIM25 inhibition of alphavirus replication. We found that TRIM25 ligase activity is absolutely required for its inhibition of diverse alphaviruses through a block in viral translation (Fig 7A–7D). Interestingly, overexpression of both TRIM25-WT and -R54P results in a dampened IFN response in our hands (Fig 7E), contrasting with the previously established role of TRIM25 in activating RIG-I signaling and implicating TRIM25 as a negative regulator of the type I IFN response [69]. Moreover, TRIM25-R54P with a complete loss of antiviral activity actually exhibits relatively more production of IFN and a subset of ISG mRNAs (Fig 7E). Still, these data together suggest that the robust TRIM25 antiviral activity against alphaviruses is not mediated through an augmented IFN response, but through its ligase activity and subsequent ubiquitination network.

Our examination of the contribution of a subset of TRIM25-R54P specific interactors to TRIM25 antiviral activity has yielded several hits, namely RTRAF (Fig 8A), NME1 (Fig 8B), and PABPC4 (Fig 8B). Though only pooled siRNA knockdown for *RTRAF* and *NME1* gave statistically significant restored viral replication, pooled siRNA knockdown of PABPC4 still restored viral replication by approximately 5 fold (Fig 8B). Additionally, both NME1 and PABPC4 expression significantly and negatively correlated with viral replication (Fig 8C–8F). RTRAF, also known as hCLE or C14orf166, is an RNA binding protein involved in cellular transcription, translation, and RNA transport, and is required for influenza virus replication [70–72]. Notably, RTRAF is a member of a cap-binding complex that activates mRNA translation [71]. Given RTRAF’s role in facilitating translation of mRNAs, it is therefore tempting to speculate that RTRAF may be required for translation of alphavirus RNA, and TRIM25-mediated ubiquitination of RTRAF may affect its ability to do so. The novel bona fide TRIM25 substrate NME1, which functions as a major synthesizer of non-ATP nucleoside triphosphates, upon ubiquitination may inhibit alphavirus replication via a similar mechanism as the potent restriction factor SAMHD1, which depletes deoxynucleotide pools, effectively preventing replication of varied DNA viruses and reverse transcription of HIV-1 [73]. On the other hand, TRIM25-mediated ubiquitination of NME1 may inhibit its metastatic suppressor activities, potentially serving as a novel mechanism for TRIM25’s previously described roles in carcinogenesis. Finally, TRIM25-mediated ubiquitination of PABPC4 could inhibit translation initiation by interfering with necessary protein-protein interactions to form the mRNA closed loop structure for ribosomal recruitment. Alternatively, it is possible that PABPC4 could inhibit alphavirus replication in a manner similar to its general block of coronavirus replication by recruiting TRIM25 to target alphavirus proteins for degradation [58]. Further studies need to be carried out to elucidate the functional consequences of these TRIM25 substrates in blocking viral translation and other cellular processes.

The novelty of this work lies within our innovative approach to uncover the multifaceted TRIM25 ubiquitination network, which is likely involved in mediating TRIM25 cellular and antiviral functions. Many questions remain unanswered as to how TRIM25-mediated ubiquitination modulates the activity of these substrates. In contrast to the more binary consequences of K48-linked dependent degradation, other types of ubiquitin linkage may effect more nuanced cellular changes by modulating substrate activity and localization [61]. Given TRIM25 proclivity for K63 linkages in the context of alphavirus infection and innate immunity [19–21], we are tempted to speculate that TRIM25 eschews a simple degradation approach in favor of a more refined modulation of substrate activity and localization. Current therapeutics that harness E3 ligases focus on their degradative power, generating compounds that bring ligases in close proximity to a target protein for degradation [74]. Further research is warranted to explore the utility of alternate modes of ubiquitination in biological therapeutics.

## Materials and methods

All resources utilized in this study are compiled below (Table 5) and referenced in the relevant methods in the following sections.

**Table 5:** Key resources.

Reagent type (species) or resource	Description	Source or reference	Identifiers	Additional information
strain (*Chikungunya virus*)	CHIKV strain 7142.181/25	[75]		
strain (*O’nyong-nyong virus*)	ONNV-GFP	[76]	GenBank AF079456	SG650 genome
strain (*Ross River virus*)	RR64-GFP	[77]		
strain (*Sindbis virus)*	Toto1101	[78]		
strain (*Sindbis virus*)	Toto1101/Luc	[79]		
strain (*Sindbis virus*)	Toto1101/Luc:ts6	[79]		
strain (*Sindbis virus*)	TE-5’2J/GFP	[80]		
strain (*Venezuelan equine encephalitis virus*)	VEEV-GFP	[81]	vaccine strain TC-83	
Antibody	anti-actin-HRP, mouse monoclonal	Sigma-Aldrich	A3854	WB (1:20,000)
Antibody	anti-EFP/TRIM25, mouse monoclonal	BD Biosciences	610570	WB (1:5,000)
Antibody	anti-FLAG, mouse monoclonal	Sigma-Aldrich	F1804	WB (1:20,000)
Antibody	anti-G3BP1, mouse monoclonal	Santa Cruz	sc-365338	WB (1:500)
Antibody	anti-G3BP2, rabbit polyclonal	Assay Biotech	C18193	WB (1:1,000)
Antibody	anti-HA, rat monoclonal	Roche Life Science	3F10	WB (1:1,000)
Antibody	anti-myc, rabbit polyclonal	Cell Signaling Technology	2272S	WB (1:2,500)
Antibody	anti-NM23A (NME1), rabbit monoclonal	Abcam	ab171935	WB (1:10,000)
Antibody	anti-PABPC4, rabbit polyclonal	Proteintech	14960-1-AP	WB (1:2,000)
Antibody	anti-UPF1, rabbit monoclonal	Cell Signaling Technology	12040	WB (1:1,000)
Antibody	anti-V5, mouse monoclonal	Invitrogen	MA5-1523	WB (1:5,000)
Antibody	donkey anti-rat HRP	Jackson ImmunoResearch	712-035-153	WB (1:20,000)
Antibody	goat anti-mouse HRP	Jackson ImmunoResearch	115-035-146	WB (1:20,000)
Antibody	goat anti-rabbit HRP	Thermo Fisher Scientific	31462	WB (1:20,000)
Chemical compound, drug	poly(I:C) HMW	InvivoGen	tlrl-pic	
Chemical compound, drug	Poly-L-lysine hydrobromide	Sigma-Aldrich	P2636	
Chemical compound, drug	Roche cOmplete Mini, EDTA-free Protease Inhibitor Cocktail	Sigma-Aldrich	11836170001	
Commercial assay or kit	DharmaFECT 1 Transfection Reagent	Horizon Discovery	T-2001-01	
Commercial assay or kit	Dynabeads Protein A for Immunoprecipitation	Invitrogen	10-002-D	
Commercial assay or kit	EZview Red ANTI-FLAG M2 Affinity Gel	Sigma-Aldrich	F2426	
Commercial assay or kit	EZview Red ANTI-MYC M2 Affinity Gel	Sigma-Aldrich	E6654	
Commercial assay or kit	KOD Hot Start Master Mix	Sigma-Aldrich	71842	
Commercial assay or kit	Luna Universal qPCR Master Mix	New England Biolabs	M3003X	
Commercial assay or kit	Mini-PROTEAN TGX Gels, 4–15%, 15 well	Bio-Rad	4568086	
Commercial assay or kit	NuPAGE MOPS SDS running buffer	Invitrogen	NP0001	
Commercial assay or kit	ProSignal Full-Range Prestained Protein Ladder	Genesee Scientific	83–650	
Commercial assay or kit	ProSignal Pico ECL Reagent	Genesee Scientific	20-300B	
Commercial assay or kit	Protoscript II First Strand cDNA Synthesis Kit	New England Biolabs	E6560L	
Commercial assay or kit	Q5 Site-Directed Mutagenesis Kit	New England Biolabs	E0552S	
Commercial assay or kit	Quick-DNA Miniprep-Plus kit	Zymo Research	D4068	
Commercial assay or kit	Quick-RNA kit	Zymo Research	R1055	
Commercial assay or kit	QuikChange II XL Site-Directed Mutagenesis Kit	Agilent	210518	
Commercial assay or kit	RNeasy mini kit	Qiagen	74104	
Commercial assay or kit	X-tremeGENE9 Transfection Reagent	Sigma-Aldrich	6365787001	
Sequence-based reagent	DNA primers for molecular cloning	This work	S1 Table	
Sequence-based reagent	RT-qPCR oligonucleotides	PrimerBank	S3 Table	https://pga.mgh.harvard.edu/primerbank/
Sequence-based reagent	siRNA	Ambion	S2 Table	
Software, algorithm	Database for Annotation, Visualization and Integrated Discovery v6.8	Frederick National Laboratory for Cancer Research, Frederick, MD		https://david.ncifcrf.gov/home.jsp
Software, algorithm	EnhancedVolcano	Clinical Bioinformatics Research LTD, United Kingdom		https://github.com/kevinblighe/EnhancedVolcano
Software, algorithm	FlowJo	BD Biosciences, Franklin, NJ		https://flowjo.com
Software, algorithm	Geneious Prime (2021.2)	Biomatters, San Diego, CA		https://geneious.com
Software, algorithm	GraphPad Prism 9 (v.9.2.0)	GraphPad Software, San Diego, CA		https://graphpad.com
Software, algorithm	ImageJ	National Institutes of Health, Bethesda, MD		https://imagej.net/
Software, algorithm	RStudio software (v.1.4.1106)	RStudio, Boston, MA		https://rstudio.com
Software, algorithm	UCSF Chimera	University of California, San Francisco, San Francisco, CA		https://www.rbvi.ucsf.edu/chimera/

### Cell culture, viruses, and infections

ZAP KO 293T cells (clone 89) and its respective parental 293T cells were generously provided by Dr. Akinori Takaoka at Hokkaido University [82]. 293T (parental, ZAP KO, and TRIM25 KO (see below) with or without inducible expression of TRIM25) were cultured in Dulbecco’s Modified Eagle Medium (DMEM; Thermo Fisher Scientific, Waltham, MA) supplemented with 10% fetal bovine serum (FBS; Avantor Seradigm, Radnor, PA). Baby hamster kidney 21 (BHK-21; American Type Culture Collection, Manasass, VA) cells were cultured in Minimal Essential Media (Thermo Fisher Scientific) supplemented with 7.5% FBS.

Wild-type SINV (Toto1101), temperature-sensitive SINV (Toto1101/Luc:ts6), SINV expressing firefly luciferase (Toto1101/Luc), SINV expressing EGFP (TE/5’2J/GFP), CHIKV vaccine strain 181/clone 25 (generously provided by Scott Weaver, The University of Texas Medical Branch at Galveston), ONNV expressing EGFP (generously provided by Dr. Steve Higgs, Kansas State University), RRV expressing EGFP (generously provided by Dr. Mark Heise, University of North Carolina), and VEEV vaccine strain TC-83 (generously provided by Dr. Ilya Frolov, University of Alabama at Birmingham) have been previously described [75–80]. Viral stocks and titers for multiplicity of infection (MOI) calculations were generated in BHK-21 cells as previously described [79]. Viral infections and plaque assays were performed as previously described [79]. TRIM25 inducible cells (see below) were induced for TRIM25 expression and infected with EGFP expressing viruses at an MOI of 0.01 plaque forming units (PFU)/cell, harvested at 10–24 hours post-infection (h.p.i.), and fixed in 1% paraformaldehyde for flow cytometry analysis. Data was acquired using a MACSQuant Analyzer 10 (Miltenyi Biotec, Auburn, CA) and analyzed using FlowJo (BD Biosciences, Franklin Lakes, NJ). Percent infected (GFP+) cells was calculated and normalized to the -dox condition of each respective cell line.

### Plasmids and transfections

Addgene plasmids for HA-tagged ubiquitin (pRK5-HA-Ubiquitin-WT, #17608; pRK5-HA-Ubiquitin-K48, #17605; pRK5-HA-Ubiquitin-K63, #17606), UPF1 (pCW57.1-Tet-UPF1WT, #99146), and PABPC4 (pDESTmycPABPC4, #19877) were used [47,83,84]. Full-length TRIM25 was generously provided by Dr. Jae U. Jung at the University of Southern California [19]. Dr. Gerald McInerney at the Karolinska Institutet, Sweden, generously provided pGFP-G3BP1 and pGFP-G3BP2a [59]. The coding sequence of NME1 isoform a (NM_198175.1) was synthesized as a gene fragment (Integrated DNA Technologies, Coralville, IA), where the ends were flanked by restriction enzyme sites NotI and XbaI, and random nucleotides were incorporated to maintain the open reading frame. Dr. Oliver Fregoso kindly gifted us a pcDNA3.1-3XFLAG plasmid. The 3XFLAG tag was swapped out for a V5 tag or a myc tag using BamHI and HindIII restriction sites to generate V5-pcDNA3.1 or myc-pcDNA3.1, respectively. The plasmid pcDNA3.1-3XFLAG was used as an expression vector for TRIM25, pcDNA3.1-V5 for UPF1, and pcDNA3.1-myc for G3BP1, G3BP2, NME1, and PABPC4. TRIM25 was cloned into pcDNA3.1-3XFLAG using XhoI and XbaI restriction sites, while UPF1, G3BP, NME1, and PABPC4 were cloned into either pcDNA3.1-V5 (UPF1) or pcDNA3.1-myc (G3BP, NME1, and PABPC4) using the NotI and XbaI restriction sites. TRIM25 RING domain mutants (I15K, R54P, I15K/R54P) were generated by mutagenesis of pcDNA-3XFLAG-TRIM25 using the QuikChange II XL Site-Directed Mutagenesis Kit (Agilent Technologies, Santa Clara, CA), while the TRIM25-PTAA mutant was generated using the Q5 Site-Directed Mutagenesis Kit (New England Biolabs, Ipswich, MA), by performing sequential mutagenesis reactions to individually mutate each residue to alanine. TRIM25 was cloned into a 3XFLAG expressing ePiggyBac transposon plasmid at the ClaI and NotI restriction sites. For cloning and mutagenesis primers, see S1 Table. All plasmids were verified by sequencing (Genewiz, South Plainfield, NJ).

Cells were transfected using X-tremeGENE9 DNA Transfection Reagent (Roche Life Science, Basel, Switzerland) at a ratio of 3 μL to1 μg DNA according to the manufacturer’s instructions. Empty vectors (pcDNA3.1-3XFLAG, V5, or myc) were transfected as necessary to keep total plasmid amount in co-transfections constant.

### *TRIM25* targeting by CRISPR

The MIT Optimized CRISPR Design portal (crispr.mit.edu) and CHOPCHOP [85] (chopchop.cbu.uib.no) were used to design guide RNAs (gRNAs) targeting exon 1 of the human *TRIM25* gene (S1A Fig). The guide with the highest ranking in both scoring programs (5’-CGGCGCAACAGGTCGCGAACGGG-3’) was selected for cloning into the PX459 vector (Addgene, #62988), a non-lentiviral construct that also delivers Cas9 [86]. Oligos containing the gRNA sequences (5’- CACCGCGGCGCAACAGGTCGCGAAC-3’ and 5’- AAACGTTCGCGACCTGTTGCGCCGC-3’) were ligated and cloned into PX459 linearized with BbsI. 293T cells were transiently transfected with PX459 expressing TRIM25 gRNA and selected with 1 μg/mL puromycin the next day to eliminate untransfected cells. Following two days of puromycin selection, surviving cells were counted, diluted to 0.3 cell/well in a 96-well plate, and seeded in 10% FBS DMEM. Single cell clones were expanded and treated with or without puromycin. Clones sensitive to puromycin, indicating failure to integrate gRNA expressing vector, were harvested for immunoblot analysis to assess TRIM25 expression. Five clones (3, 6, 8, 9, and 10) were selected based on western blotting results indicating complete loss of TRIM25 protein expression (S1B Fig). Viral replication within these clones was characterized by infection with a luciferase-expressing SINV (Toto1101/Luc). Clone #8 was selected for generation of TRIM25 inducible cell lines based on its intermediate viral replication phenotype (S1C Fig), similar to previous TRIM25 siRNA data [20]. A 600-bp amplicon flanking the gRNA targeting site was amplified from genomic DNA isolated from each clonal population using a Quick-DNA Miniprep Plus kit (Zymo Research, Irvine, CA) and KOD Hot Start Master Mix (Millipore Sigma). Amplicons from clone #8 were sent to Massachusetts General Hospital Center for Computational and Integrative Biology DNA Core for Complete Amplicon Sequencing, confirming that CRISPR targeting results in deletions in exon 1 of TRIM25, leading to frameshift mutations and premature stop codons in both alleles (S1D Fig).

### Generation of TRIM25 inducible cell lines

To reconstitute TRIM25 expression (WT and R54P) in our TRIM25 KO 293T cell line (clone #8; see above for details), we used the enhanced PiggyBac (ePB) transposable element system provided by the Brivanlou laboratory at the Rockefeller University, as previously described [87,88]. TRIM25 KO 293T cells were transfected with 1:1 ePB transposon vector encoding TRIM25-WT or TRIM25-R54P and the transposase plasmid. Two days post-transfection, 1.5 μg/mL of puromycin was used to select a population of TRIM25 KO 293T cells inducible for TRIM25-WT, -R54P, or -PTAA, which were then expanded and treated with different amounts of dox (0.001, 0.01, 0.1, 1, and 10 μg/mL) to confirm TRIM25 inducible expression by immunoblotting.

### Mass spectrometry (MS)

To identify TRIM25 substrates, three 15-cm dishes per condition were seeded with 7.5x10^6^ TRIM25 inducible or TRIM25 KO 293T cells each in the presence of 1 μg/mL dox. Two days later, cells were mock infected or infected with Toto1101 at an MOI of 1 PFU/cell. Six hours post infection, cells were trypsinized, spun down, and lysed in 3 mL of FLAG IP buffer. Supernatant was transferred to a new 15 mL tube and supplemented with 5 mL of FLAG IP buffer before incubating with 80 μL of anti-FLAG beads for 45 min at 4°C, rotating. Immunoprecipitates were washed three times in FLAG IP buffer before elution with 130 μL of 8M urea in 100 mM Tris-HCl, pH 8, shaken for 10 min at 1200 rpm. Supernatant was carefully transferred to a new tube and proteins were precipitated by addition of 4 volumes of -20°C acetone and incubation at 4°C overnight. After centrifugation at 16,100 g for 30 min at 4°C, pellets were washed with -20°C acetone and centrifuged again.

Dried pellets were processed at the UCLA Proteomics Core. Protein samples were reduced and alkylated using 5mM Tris (2-carboxyethyl) phosphine and 10mM iodoacetamide, respectively, and then proteolyzed by the sequential addition of trypsin and lys-C proteases at 37°C as described [89]. Digested peptides were resuspended in 5% formic acid and fractionated online using a 25cm long, 75 μM inner diameter fused silica capillary packed in-house with bulk C18 reversed phase resin (length, 25 cm; inner diameter, 75 μM; particle size, 1.9 μm; pore size, 100 Å; Dr. Maisch GmbH) [90]. The 140 min water-acetonitrile gradient was delivered using a Dionex Ultimate 3000 UHPLC system (Thermo Fisher Scientific) at a flow rate of 300 nL/min (Buffer A: water with 3% DMSO and 0.1% formic acid and Buffer B: acetonitrile with 3% DMSO and 0.1% formic acid). Fractionated peptides were ionized and analyzed by tandem mass spectrometry (MS/MS) Orbitrap Fusion Lumos mass spectrometer (Thermo Fisher Scientific). Label-free quantitation was performed using the MaxQuant software package [91]. The mass spectrometry proteomics data have been deposited to the ProteomeXchange Consortium via the PRIDE [92] partner repository with the dataset identifier PXD034024. The EMBL Human reference proteome (UP000005640 9606) was utilized for all database searches. Statistical analysis of MaxQuant output data was performed with the artMS Bioconductor [93] package which performs the relative quantification of protein abundance using the MSstats Bioconductor package (default parameters). Intensities were normalized across samples by median-centering the log2-transformed MS1 intensity distributions. The abundance of proteins missing from one condition but found in more than 2 biological replicates of the other condition for any given comparison were estimated by imputing intensity values from the lowest observed MS1-intensity across samples and p-values were randomly assigned to those between 0.05 and 0.01 for illustration purposes. Significant hits were defined as interactors that possessed a log2FoldChange of >1.5 and a -log10Pvalue > 1.3.

### TRIM25 autoubiquitination and co-immunoprecipitation (co-IP) assay

To assess TRIM25 autoubiquitination or co-IP with proteins of interest, transfected or untransfected cells in 6-well plates were collected and lysed by rotating for 30 min at 4°C in FLAG IP buffer (100 mM Tris-HCl 8.0, 150 mM NaCl, 5 mM EDTA, 1 mM DTT, 5% glycerol, 0.1% NP-40) supplemented with a complete protease inhibitor cocktail (Roche Life Science), before spinning down at 14000 rpm for 15 min at 4°C. Anti-FLAG beads (EZview Red ANTI-FLAG M2 Affinity Gel, Sigma-Aldrich, St. Louis, MO) or anti-myc beads (EZview Red ANTI-MYC M2 Affinity Gel, Sigma-Aldrich) were equilibrated by washing 3 times in FLAG IP buffer. Three hundred μL of whole cell lysate (WCL) were incubated with 30 μL of anti-FLAG beads for 45 min at 4°C, rotating. Immunoprecipitates were washed 3 times with the FLAG IP buffer. Bound proteins were eluted with SDS loading buffer and boiled for 5 minutes for immunoblot analysis.

### Ubiquitination IP assay

To assess TRIM25 ubiquitination of putative substrates, immunoprecipitation was performed essentially as previously described [20]. Briefly, cells were collected and lysed in 0.5% SDS buffer supplemented with complete protease inhibitor cocktail. Three hundred μL of WCL were diluted into 1X TNA buffer (0.25% Triton, 50 mM Tris-HCl, pH 7.5; 200 mM NaCl, 1 mM EDTA) + 2 mg/mL BSA. WCL containing V5-tagged substrates were then incubated with 1 μg of anti-V5 antibody overnight at 4°C. The next morning, 40 μL Protein A Dynabeads (Invitrogen, Waltham, MA) were added and incubated for 2 h at 4°C. WCL containing myc-tagged substrates were incubated directly with anti-myc beads for 45 minutes at 4°C, rotating. Following incubation with beads, both myc-tagged and V5-tagged immunoprecipitates were washed 3 times with 1X TNA buffer + 2 mg/mL BSA. Myc-tagged NME1 underwent an additional two washes with 1X TNA buffer only. Bound proteins were eluted with SDS loading buffer and boiled for 5 minutes for immunoblot analysis.

### Immunoblot analysis

Proteins were resolved through SDS-PAGE using 4–15% precast Mini-PROTEAN TGX Gels (Bio-Rad) and NuPAGE MOPS SDS running buffer (Thermo Fisher Scientific) before transferring to a PVDF membrane (Bio-Rad). Immunodetection was achieved with 1:5,000 anti-ZAP (Abcam, Cambridge, United Kingdom); 1:5,000 anti-TRIM25 (BD Biosciences), 1:1,000 anti-HA (Roche Life Science), 1:5000 anti-V5 (Invitrogen), 1:2,500 anti-myc (Cell Signaling Technology, Danvers, MA), 1:20,000 anti-FLAG (Sigma-Aldrich), 1:500 anti-G3BP1 (Santa Cruz, Dallas, TX), 1:1,000 anti-G3BP2 (Assay Biotech, Fremont, CA), 1:1,000 anti-UPF1 (Cell Signaling Technology), 1:10,000 anti-NME1 (Abcam), 1:2,000 anti-PABPC4 (Proteintech, Rosemont, IL), and 1:20,000 anti-actin-HRP (Sigma-Aldrich). Primary antibodies were detected with 1:20,000 goat anti-mouse HRP (Jackson ImmunoResearch, West Grove, PA), 1:20,000 goat anti-rabbit HRP (Thermo Fisher Scientific), or 1:20,000 donkey anti-rat HRP (Jackson ImmunoResearch). Proteins were resolved on a 4–15% Mini-PROTEAN TGX gel (Bio-Rad, Hercules, CA) and visualized using ProSignal Pico ECL Reagent (Genesee Scientific, San Diego, CA) on a ChemiDoc (Bio-Rad). Quantification of western blots was performed using ImageJ.

### siRNA knockdown and poly(I:C) stimulation

Ambion Silencer siRNAs (S2 Table) and nontargeting controls (Thermo Fisher Scientific) were reverse transfected with DharmaFECT 1 Transfection Reagent (Horizon Discovery, Cambridge, United Kingdom) according to manufacturer protocols. Briefly, siRNAs were mixed with DharmaFECT 1 Transfection Reagent (1:100 dilution in HBSS) and 50 μL of siRNA mix were added to each well in a 24 well plate, or 100 μL in a 12 well plate. 1.2 x 10^5^ cells were added per well in 250 μL in a 24 well plate or 2.4 x 10^6^ in 500 μL in a 12 well plate, for a final concentration of 25 nM siRNA. Plates that would be subjected to SINV infection were first poly-L-lysine treated. Cells were induced for TRIM25 expression using a final concentration of 1 μg/mL dox one day post-transfection, as applicable. Cells were harvested for RNA extraction for RT-qPCR to quantify gene knockdown or subjected to SINV infection 48 h post-transfection. To assess ISG induction in TRIM25 inducible cells upon poly(I:C) treatment, cells were treated with 1 μg poly(I:C) HMW (InvivoGen, San Diego, CA) in the presence or absence of 1 μg/mL dox per well, and harvested for RNA extraction for RT-qPCR.

### Quantitative reverse transcription PCR (RT-qPCR)

Total RNA was isolated from siRNA- and poly(I:C)-treated cells using the RNeasy mini kit (Qiagen, Hilden, Germany) or the Quick-RNA kit (Zymo Research). 400 ng to 1 μg of input RNA was used as a template for reverse transcription using Protoscript II First Strand cDNA Synthesis Kit (New England Biolabs) and random hexamers, following manufacturer instructions. RT-qPCR was performed using 5 μL of 4 to 10-fold-diluted cDNA, and Luna Universal qPCR Master Mix (New England Biolabs) in the CFX Real-Time PCR system (Bio-Rad), courtesy of the UCLA Virology Core. qPCR conditions were as follows: initial denaturation step at 95°C for 1 min, then 40 cycles of 95°C for 15 sec followed by 60°C for 30 sec, concluding with a final 10 sec at 60°C. A melt curve was then calculated by heating to 95°C incrementally by 0.5°C/s for 10 sec at each temperature. Transcript levels of R54P specific interactors and ISGs were determined by normalizing the target transcript CT value to the CT value of the RPS11 transcript, an endogenous housekeeping gene. Fold change was calculated using this normalized value relative to the average of cells treated with the NT siRNA control or the -dox condition for respective cell lines (CT method). For RT-qPCR primers, see S3 Table.

### Statistical analysis

Statistical analyses in Figs 7 and 8, and S2 were performed on biological replicates from triplicate wells, unless indicated otherwise, using GraphPad Prism. Spearman’s rho was calculated using Microsoft Excel. For statistical analyses and numerical data underlying graphical depictions, see S1 Data.

## Supporting information

S1 Data Excel spreadsheet containing, in separate sheets, the underlying numerical data and statistical analysis for Fig panels 7A, 7B, 7C, 7D, 7E, 8A, 8B, 8C, 8D, 8E, 8F, S1C, S2A, S2B, S2C, S2D, S2E, S2F, S3A, S3B, S4A, S4B, and for Spearman’s rho calculations for NME1 and PABPC4. (XLSX)

S1 Fig Validation of TRIM25 KO in CRISPR clones. (A) Schematic of where TRIM25 sgRNA targets exon 1. (B) Western blot of TRIM25 KO CRISPR single cell clones. (C) Cells were infected with SINV Toto1101/Luc at an MOI of 0.01 PFU/cell and lysed at 6, 12, 24, and 40 h.p.i. for measurement of luciferase activity. (D) CRISPR-targeting region in the genomic sequence of TRIM25 is shown in clone 8. The alignment shown is in the same reading frame of the wild-type TRIM25 protein. A red dash represents a deletion when compared to the wild-type TRIM25 sequence.
(TIF)

S2 Fig G3BP and UPF1 are not sufficient to mediate TRIM25 antiviral activity. (A-C) TRIM25- inducible cells were induced for TRIM25-WT, -R54P, or -PTAA expression at 1 μg/mL dox, infected with (A) SINV Toto1101/Luc at an MOI of 0.01 PFU/cell, and lysed at 6, 12, 24, 32, and 40 h.p.i.; or with (B) SINV Toto1101/Luc:ts6 at an MOI of 1 PFU/cell and lysed at 0, 2, 4, and 6 h.p.i. for measurement of luciferase activity; or (C) CHIKV at an MOI of 0.01 PFU/cell, harvesting supernatant at 6 and 24 h.p.i. for plaque assays. Open circles and dashed lines indicate absence of TRIM25 induction. Data are representative of two independent experiments. Error bars represent (A-B) range or (C) standard deviation. Asterisks indicate statistically significant differences (Two-way ANOVA and Tukey’s multiple comparisons test: **, p<0.01; ***, p<0.001; ****, p<0.0001). Light blue compares WT +/- dox, dark blue for R54P +/- dox, and green for PTAA +/- dox. Unlabeled comparisons are not significant. (D) TRIM25-WT inducible cells were transfected with NT pool siRNA or UPF1 siRNAs in the absence of dox. RNA was extracted 48 hours post-transfection for RT-qPCR analysis. Data are combined from two independent experiments. (E-F) TRIM25-WT inducible cells were transfected with NT pool siRNA or UPF1 siRNA #1, induced for TRIM25-WT expression at 1 μg/mL dox, and infected with Toto1101/Luc at an MOI of 0.01 PFU/cell. Cells were lysed at 24 h.p.i. for (E) measurement of luciferase activity or (F) quantification of UPF1 knockdown via RT-qPCR. Data are combined from three independent experiments. Asterisks indicate statistically significant differences (Two-way ANOVA and Šídák’s multiple comparisons test: ****, p<0.0001). Unlabeled comparisons are not significant.
(TIF)

S3 Fig Poly(I:C) treatment robustly induces *IFN-β* mRNA expression. (A-B) TRIM25 inducible cells were treated with poly(I:C) in the presence or absence of (A) TRIM25-WT or (B) TRIM25-R54P induction. RNA was harvested for RT-qPCR analysis. Data are representative of two independent experiments.
(TIF)

S4 Fig Validation of pooled siRNA knockdown. (A-B) TRIM25 inducible cells were transfected with pooled siRNAs for either (A) hits specific to TRIM25-R54P in the absence of viral infection or (B) hits specific to TRIM25-R54P in the presence of viral infection. Cells were induced for TRIM25-WT expression at 1 μg/mL dox. RNA was extracted for RT-qPCR analysis.
(TIF)

S1 Table Cloning and mutagenesis primers. (DOCX)

S2 Table siRNAs. (DOCX)

S3 Table RT-qPCR primers. (DOCX)
