# Supplementary figures and images for: Elucidation of TRIM25 ubiquitination targets involved in diverse cellular and antiviral processes
Source: PLoS Pathog. 2022 Sep 6;18(9):e1010743. doi: 10.1371/journal.ppat.1010743 (PMC9481182; doi:10.1371/journal.ppat.1010743)

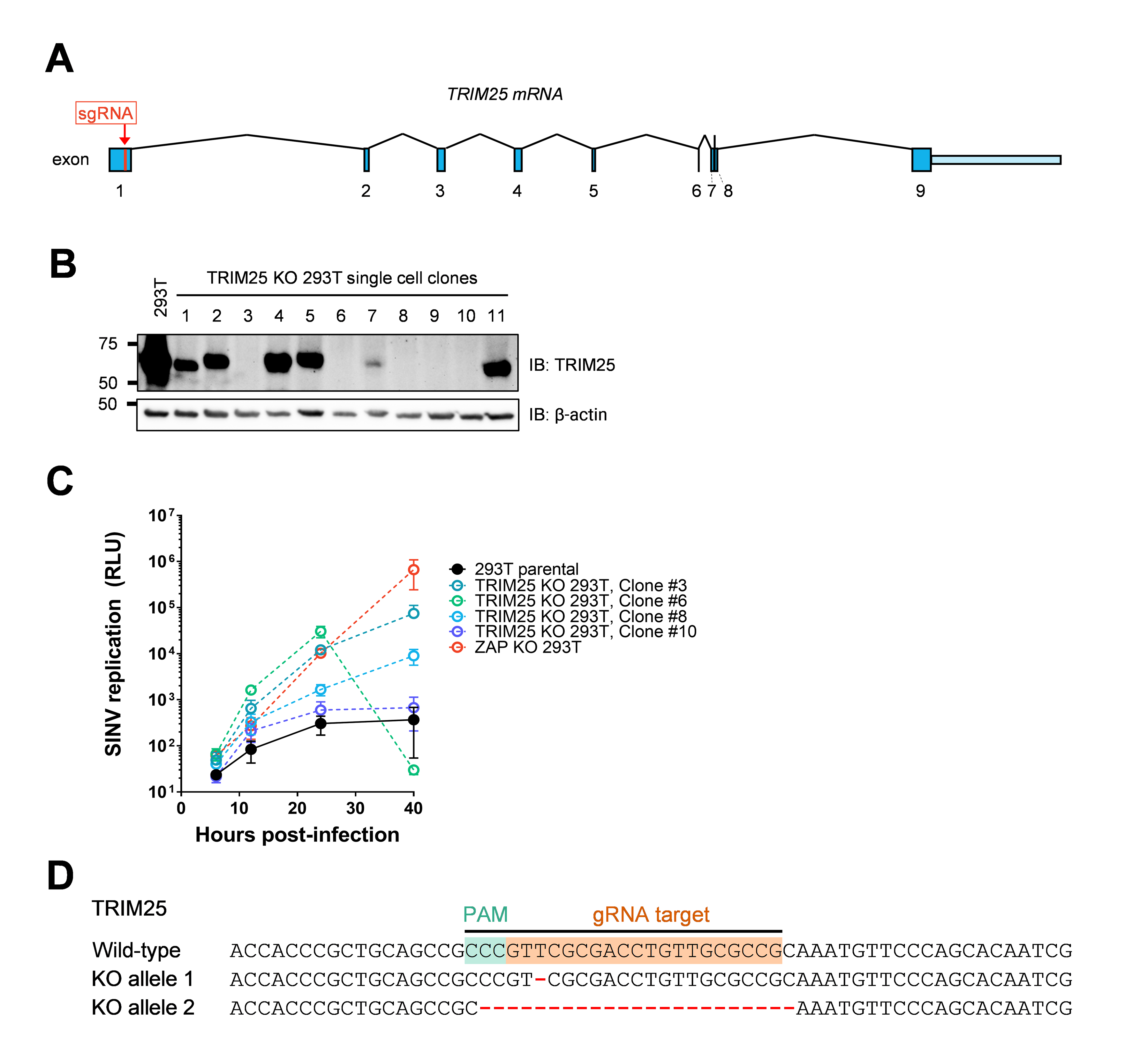

Supplement: S1 Fig — (A) Schematic of where TRIM25 sgRNA targets exon 1. (B) Western blot of TRIM25 KO CRISPR single cell clones. (C) Cells were infected with SINV Toto1101/Luc at an MOI of 0.01 PFU/cell and lysed at 6, 12, 24, and 40 h.p.i. for measurement of luciferase activity. (D) CRISPR-targeting region in the genomic sequence of TRIM25 is shown in clone 8. The alignment shown is in the same reading frame of the wild-type TRIM25 protein. A red dash represents a deletion when compared to the wild-type TRIM25 sequence. (TIF) [file ppat.1010743.s002.tif]

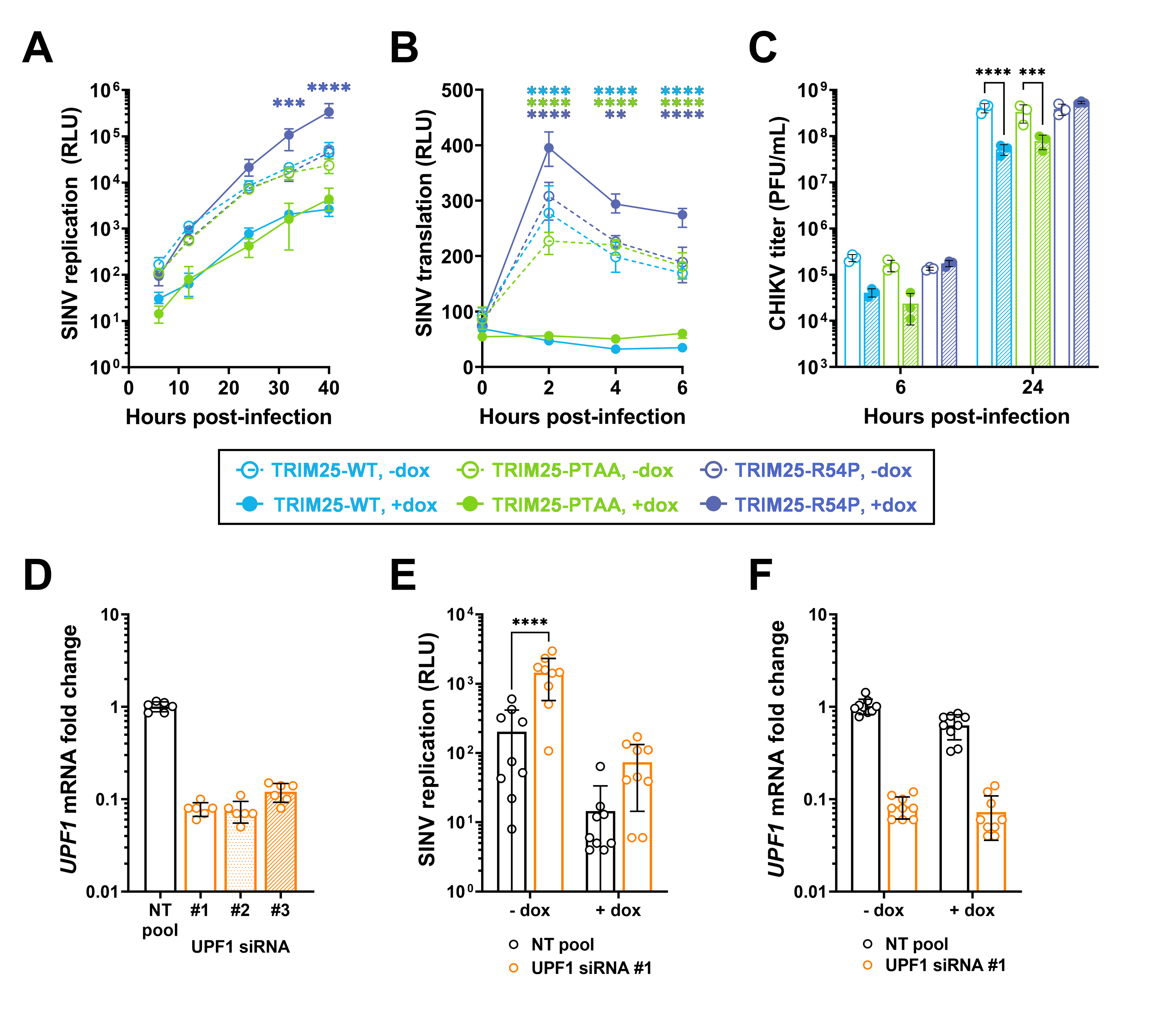

Supplement: S2 Fig — (A-C) TRIM25- inducible cells were induced for TRIM25-WT, -R54P, or -PTAA expression at 1 μg/mL dox, infected with (A) SINV Toto1101/Luc at an MOI of 0.01 PFU/cell, and lysed at 6, 12, 24, 32, and 40 h.p.i.; or with (B) SINV Toto1101/Luc:ts6 at an MOI of 1 PFU/cell and lysed at 0, 2, 4, and 6 h.p.i. for measurement of luciferase activity; or (C) CHIKV at an MOI of 0.01 PFU/cell, harvesting supernatant at 6 and 24 h.p.i. for plaque assays. Open circles and dashed lines indicate absence of TRIM25 induction. Data are representative of two independent experiments. Error bars represent (A-B) range or (C) standard deviation. Asterisks indicate statistically significant differences (Two-way ANOVA and Tukey’s multiple comparisons test: **, p<0.01; ***, p<0.001; ****, p<0.0001). Light blue compares WT +/- dox, dark blue for R54P +/- dox, and green for PTAA +/- dox. Unlabeled comparisons are not significant. (D) TRIM25-WT inducible cells were transfected with NT pool siRNA or UPF1 siRNAs in the absence of dox. RNA was extracted 48 hours post-transfection for RT-qPCR analysis. Data are combined from two independent experiments. (E-F) TRIM25-WT inducible cells were transfected with NT pool siRNA or UPF1 siRNA #1, induced for TRIM25-WT expression at 1 μg/mL dox, and infected with Toto1101/Luc at an MOI of 0.01 PFU/cell. Cells were lysed at 24 h.p.i. for (E) measurement of luciferase activity or (F) quantification of UPF1 knockdown via RT-qPCR. Data are combined from three independent experiments. Asterisks indicate statistically significant differences (Two-way ANOVA and Šídák’s multiple comparisons test: ****, p<0.0001). Unlabeled comparisons are not significant. (TIF) [file ppat.1010743.s003.tif]

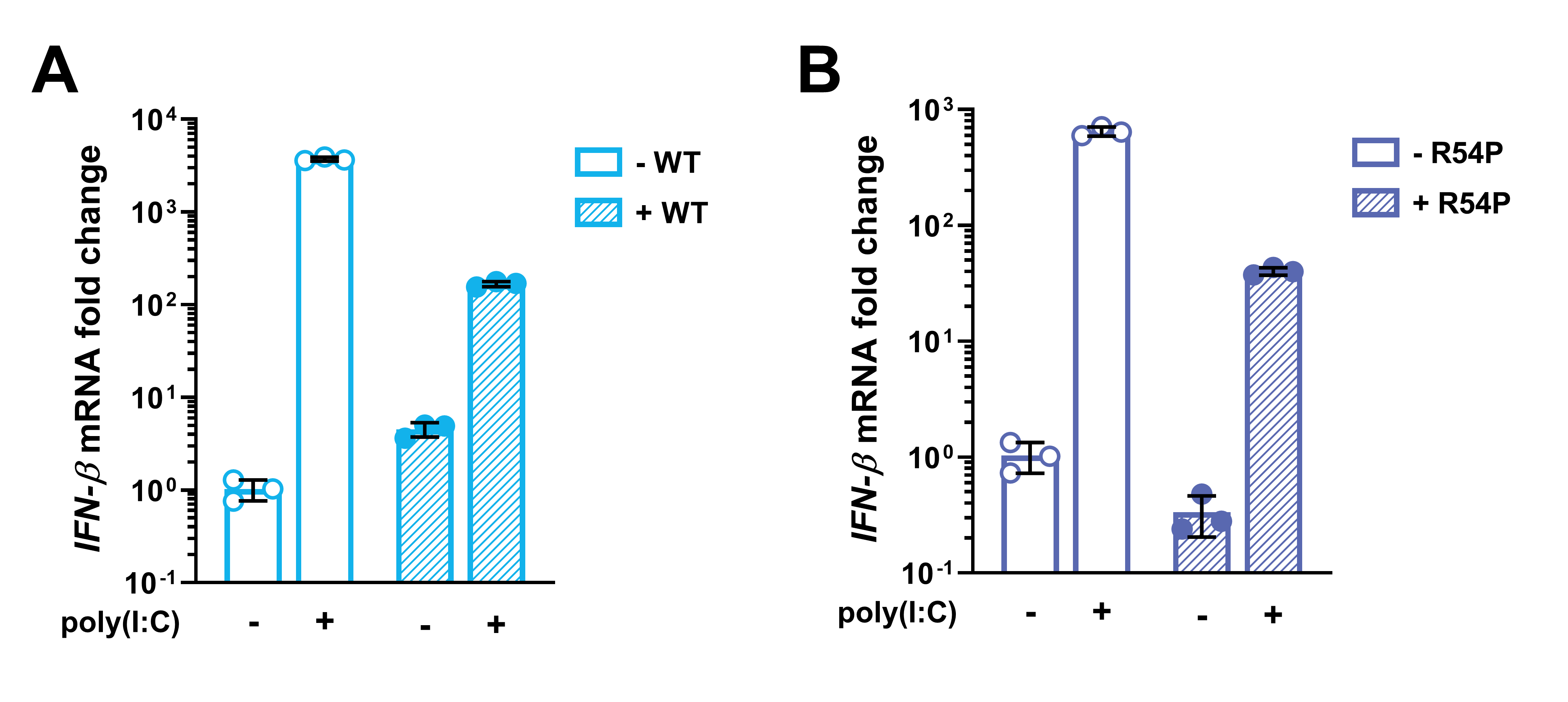

Supplement: S3 Fig — (A-B) TRIM25 inducible cells were treated with poly(I:C) in the presence or absence of (A) TRIM25-WT or (B) TRIM25-R54P induction. RNA was harvested for RT-qPCR analysis. Data are representative of two independent experiments. (TIF) [file ppat.1010743.s004.tif]

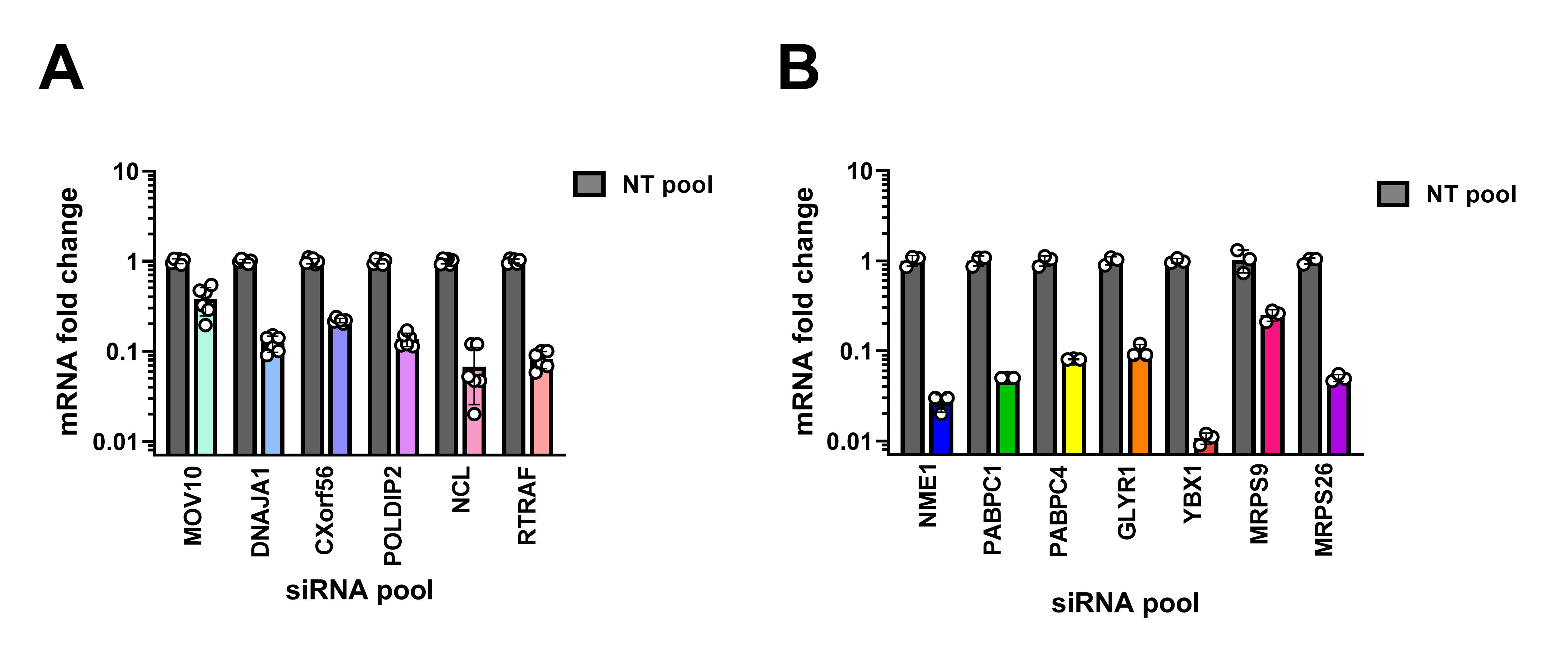

Supplement: S4 Fig — (A-B) TRIM25 inducible cells were transfected with pooled siRNAs for either (A) hits specific to TRIM25-R54P in the absence of viral infection or (B) hits specific to TRIM25-R54P in the presence of viral infection. Cells were induced for TRIM25-WT expression at 1 μg/mL dox. RNA was extracted for RT-qPCR analysis. (TIF) [file ppat.1010743.s005.tif]
