# Supplementary material for: Elucidation of TRIM25 ubiquitination targets involved in diverse cellular and antiviral processes
Source: PLoS Pathog. 2022 Sep 6;18(9):e1010743. doi: 10.1371/journal.ppat.1010743 (PMC9481182; doi:10.1371/journal.ppat.1010743)
Supplement: S1 Table — (DOCX) [file ppat.1010743.s006.docx]

| **Name** | **Primer sequence (5'-3')** | **Construction** |
| --- | --- | --- |
| XhoI-ClaI-TRIM25 F1 (EY-47) | gtttCTCGAGgtATCGATATGGCAGAGCTGTGCCCC | pcDNA-3XFLAG-TRIM25, ePiggyBac-3XFLAG-TRIM25 |
| XbaI-NotI-TRIM25 R1 (EY-48) | gtttTCTAGAGCGGCCGCCTACTTGGGGGAGCAGATGGAG | pcDNA-3XFLAG-TRIM25, ePiggyBac-3XFLAG-TRIM25 |
| TRIM25-I15K F1 (EY-10) | AGGAGCTGTCGTGCTCCAagTGCCTGGAGC | pcDNA-3XFLAG-TRIM25-I15K |
| TRIM25-I15K R1 (EY-11) | GCTCCAGGCActTGGAGCACGACAGCTCCT | pcDNA-3XFLAG-TRIM25-I15K |
| TRIM25-R54P F1 (EY-12) | CCCGCAGTGCCcCGCCGTCTACC | pcDNA-3XFLAG-TRIM25-R54P |
| TRIM25-R54P R1 (EY-13) | GGTAGACGGCGgGGCACTGCGGG | pcDNA-3XFLAG-TRIM25-R54P |
| TRIM25-PTAG F1 (EY-172) | GCTTCCCACGgcTGGAGCCCCG | pcDNA-3XFLAG-TRIM25-PTAA |
| TRIM25-PTAA F1 (EY-173) | CCCACGGCTGcAGCCCCGGAA | pcDNA-3XFLAG-TRIM25-PTAA |
| TRIM25-PTAA R2 (EY-174) | AAGCTTGCTGGGTAAGGCAGGG | pcDNA-3XFLAG-TRIM25-PTAA |
| NotI-UPF1 F1 (EY-91) | gtttGCGGCCGCaGTGGAAGCCTATGGGCC | pcDNA-V5-UPF1 |
| XbaI-UPF1 R1 (EY-92) | gtttTCTAGAGCCACGTTGCTTAGCTCTTC | pcDNA-V5-UPF1 |
| NotI-G3BP1 F1 (EY-119) | gtttGCGGCCGCaGTGATGGAGAAGCCTAGTCCCC | pcDNA-myc-G3BP1 |
| XbaI-G3BP1 R1 (EY-120) | gtttTCTAGATTACTGCCGTGGCGCAAG | pcDNA-myc-G3BP1 |
| NotI-G3BP2 F1 (EY-121 | gtttGCGGCCGCaGTTATGCAGAAGCCCAGTCCG | pcDNA-myc-G3BP2 |
| XbaI-G3BP2 R1 (EY-97) | gtttTCTAGAtcagcgacgctgtcctgtg | pcDNA-myc-G3BP2 |
| NotI-NME1 F1 (SH-08) | gtctGCGGCCGCTGTGCTACTGTCTACTTTAGG | pcDNA-myc-NME1 |
| XbaI-NME1 R1 (SH-09) | ggtaTCTAGATTATTCATAGATCCAGTTCTGAGC | pcDNA-myc-NME1 |
| NotI-PABPC4 F1 (SH-45) | gtttGCGGCCGCtAACGCTGCGGCCAG | pcDNA-myc-PABPC4 |
| XbaI-PABPC4 R1 (SH-46) | ggtaTCTAGACTAAGAGGTAGCAGCAGCAACA | pcDNA-myc-PABPC4 |

**S1 Table. Cloning and mutagenesis primers.**
