# Supplementary material for: Elucidation of TRIM25 ubiquitination targets involved in diverse cellular and antiviral processes
Source: PLoS Pathog. 2022 Sep 6;18(9):e1010743. doi: 10.1371/journal.ppat.1010743 (PMC9481182; doi:10.1371/journal.ppat.1010743)
Supplement: S2 Table — (DOCX) [file ppat.1010743.s007.docx]

| **Gene Symbol** | **Full Gene Name** | **Gene ID** | **siRNA ID** | **Exon(s) Targeted** | **Sense siRNA Sequence** |
| --- | --- | --- | --- | --- | --- |
| CXorf56 | chromosome X open reading frame 56 | [63932](http://www.ncbi.nlm.nih.gov/entrez/query.fcgi?db=gene&cmd=Retrieve&dopt=Graphics&list_uids=63932) | 29642 | 3 | GGCAUUGAACGACAGUACAtt |
| CXorf56 | chromosome X open reading frame 56 | [63932](http://www.ncbi.nlm.nih.gov/entrez/query.fcgi?db=gene&cmd=Retrieve&dopt=Graphics&list_uids=63932) | 29736 | 3 | GGAAGAAAUGUGCAAAGUGtt |
| CXorf56 | chromosome X open reading frame 56 | [63932](http://www.ncbi.nlm.nih.gov/entrez/query.fcgi?db=gene&cmd=Retrieve&dopt=Graphics&list_uids=63932) | 127302 | 7 | GCAUUUAGAUCAGGAGGCAtt |
| DNAJA1 | DnaJ (Hsp40) homolog, subfamily A, member 1 | [3301](http://www.ncbi.nlm.nih.gov/entrez/query.fcgi?db=gene&cmd=Retrieve&dopt=Graphics&list_uids=3301) | 10092 | 2 | GGAGAGAAGUUUAAACAGAtt |
| DNAJA1 | DnaJ (Hsp40) homolog, subfamily A, member 1 | [3301](http://www.ncbi.nlm.nih.gov/entrez/query.fcgi?db=gene&cmd=Retrieve&dopt=Graphics&list_uids=3301) | 10180 | 3 | GGGAAUUAUAUGACAAAGGtt |
| DNAJA1 | DnaJ (Hsp40) homolog, subfamily A, member 1 | [3301](http://www.ncbi.nlm.nih.gov/entrez/query.fcgi?db=gene&cmd=Retrieve&dopt=Graphics&list_uids=3301) | 144744 | 5 | GCGGAUCAGUCCUAAAGAUtt |
| GLYR1 | glyoxylate reductase 1 homolog | [84656](http://www.ncbi.nlm.nih.gov/entrez/query.fcgi?db=gene&cmd=Retrieve&dopt=Graphics&list_uids=84656) | 109777 | 15 | GGAUCUCCGCUUAGCCAUUtt |
| GLYR1 | glyoxylate reductase 1 homolog | [84656](http://www.ncbi.nlm.nih.gov/entrez/query.fcgi?db=gene&cmd=Retrieve&dopt=Graphics&list_uids=84656) | 109778 | 15 | GGUGUACAAAAGAGCCAAGtt |
| GLYR1 | glyoxylate reductase 1 homolog | [84656](http://www.ncbi.nlm.nih.gov/entrez/query.fcgi?db=gene&cmd=Retrieve&dopt=Graphics&list_uids=84656) | 113776 | 4 | GCCAUAUCAUGCUCAUAAAtt |
| MOV10 | Mov10, Moloney leukemia virus 10, homolog (mouse) | [4343](http://www.ncbi.nlm.nih.gov/entrez/query.fcgi?db=gene&cmd=Retrieve&dopt=Graphics&list_uids=4343) | 28823 | 2 | GGGCUGAGUAUCUUCAUGGtt |
| MOV10 | Mov10, Moloney leukemia virus 10, homolog (mouse) | [4343](http://www.ncbi.nlm.nih.gov/entrez/query.fcgi?db=gene&cmd=Retrieve&dopt=Graphics&list_uids=4343) | 28919 | 4 | GGAGCCGGCACAUUCUACAtt |
| MOV10 | Mov10, Moloney leukemia virus 10, homolog (mouse) | [4343](http://www.ncbi.nlm.nih.gov/entrez/query.fcgi?db=gene&cmd=Retrieve&dopt=Graphics&list_uids=4343) | 29008 | 20 | GGAAGAAAACUCUGAAAACtt |
| MRPS26 | mitochondrial ribosomal protein S26 | [64949](http://www.ncbi.nlm.nih.gov/entrez/query.fcgi?db=gene&cmd=Retrieve&dopt=Graphics&list_uids=64949) | 32714 | 3 | GGAAGAGGUGAAAAACUUCtt |
| MRPS26 | mitochondrial ribosomal protein S26 | [64949](http://www.ncbi.nlm.nih.gov/entrez/query.fcgi?db=gene&cmd=Retrieve&dopt=Graphics&list_uids=64949) | 130988 | 4 | CCAUGUAUGUAUCAUGGCGtt |
| MRPS26 | mitochondrial ribosomal protein S26 | [64949](http://www.ncbi.nlm.nih.gov/entrez/query.fcgi?db=gene&cmd=Retrieve&dopt=Graphics&list_uids=64949) | 130989 | 4 | GGAAGGUUCAGCCUUAUCCtt |
| MRPS9 | mitochondrial ribosomal protein S9 | [64965](http://www.ncbi.nlm.nih.gov/entrez/query.fcgi?db=gene&cmd=Retrieve&dopt=Graphics&list_uids=64965) | 131876 | 6 | CCAGAGACGUGAUUGGCAGtt |
| MRPS9 | mitochondrial ribosomal protein S9 | [64965](http://www.ncbi.nlm.nih.gov/entrez/query.fcgi?db=gene&cmd=Retrieve&dopt=Graphics&list_uids=64965) | 131877 | 10 | GCAAUACGACUGGCAAUGGtt |
| MRPS9 | mitochondrial ribosomal protein S9 | [64965](http://www.ncbi.nlm.nih.gov/entrez/query.fcgi?db=gene&cmd=Retrieve&dopt=Graphics&list_uids=64965) | 131878 | 10 | GCUGGACUACUUACUACUGtt |
| NCL | nucleolin | [4691](http://www.ncbi.nlm.nih.gov/entrez/query.fcgi?db=gene&cmd=Retrieve&dopt=Graphics&list_uids=4691) | 16052 | 2 | GGAGGUAGAAGAAGAUAGUtt |
| NCL | nucleolin | [4691](http://www.ncbi.nlm.nih.gov/entrez/query.fcgi?db=gene&cmd=Retrieve&dopt=Graphics&list_uids=4691) | 16225 | 7 | GGAAAAGACAGUAAGAAAGtt |
| NCL | nucleolin | [4691](http://www.ncbi.nlm.nih.gov/entrez/query.fcgi?db=gene&cmd=Retrieve&dopt=Graphics&list_uids=4691) | 144014 | 2 | GGUCGUCAUACCUCAGAAGtt |
| NME1 | NME/NM23 nucleoside diphosphate kinase 1 | [4830](http://www.ncbi.nlm.nih.gov/entrez/query.fcgi?db=gene&cmd=Retrieve&dopt=Graphics&list_uids=4830) | 41 | 2,3 | GGAUUCCGCCUUGUUGGUCtt |
| NME1 | NME/NM23 nucleoside diphosphate kinase 1 | [4830](http://www.ncbi.nlm.nih.gov/entrez/query.fcgi?db=gene&cmd=Retrieve&dopt=Graphics&list_uids=4830) | 42 | 3,4 | GGAACACUACGUUGACCUGtt |
| NME1 | NME/NM23 nucleoside diphosphate kinase 1 | [4830](http://www.ncbi.nlm.nih.gov/entrez/query.fcgi?db=gene&cmd=Retrieve&dopt=Graphics&list_uids=4830) | 43 | 5,6 | GGCUGUAGGAAAUCUAGUUtt |
| PABPC1 | poly(A) binding protein, cytoplasmic 1 | [26986](http://www.ncbi.nlm.nih.gov/entrez/query.fcgi?db=gene&cmd=Retrieve&dopt=Graphics&list_uids=26986) | 11745 | 8 | GGUUACUUCAUGGCAGCUAtt |
| PABPC1 | poly(A) binding protein, cytoplasmic 1 | [26986](http://www.ncbi.nlm.nih.gov/entrez/query.fcgi?db=gene&cmd=Retrieve&dopt=Graphics&list_uids=26986) | 11834 | 15 | GGGACCAUGAAAAGAAACUtt |
| PABPC1 | poly(A) binding protein, cytoplasmic 1 | [26986](http://www.ncbi.nlm.nih.gov/entrez/query.fcgi?db=gene&cmd=Retrieve&dopt=Graphics&list_uids=26986) | 216913 | 8 | GCUGUUCCCAACCCUGUAAtt |
| PABPC4 | poly(A) binding protein, cytoplasmic 4 (inducible form) | [8761](http://www.ncbi.nlm.nih.gov/entrez/query.fcgi?db=gene&cmd=Retrieve&dopt=Graphics&list_uids=8761) | 13771 | 1 | GGCCAUGCUGUACGAAAAGtt |
| PABPC4 | poly(A) binding protein, cytoplasmic 4 (inducible form) | [8761](http://www.ncbi.nlm.nih.gov/entrez/query.fcgi?db=gene&cmd=Retrieve&dopt=Graphics&list_uids=8761) | 13867 | 2 | GGGAAAGCCAAUCCGCAUCtt |
| PABPC4 | poly(A) binding protein, cytoplasmic 4 (inducible form) | [8761](http://www.ncbi.nlm.nih.gov/entrez/query.fcgi?db=gene&cmd=Retrieve&dopt=Graphics&list_uids=8761) | 13959 | 6 | GGAGAGAAUUAGUCGAUAUtt |
| POLDIP2 | polymerase (DNA-directed), delta interacting protein 2 | [26073](http://www.ncbi.nlm.nih.gov/entrez/query.fcgi?db=gene&cmd=Retrieve&dopt=Graphics&list_uids=26073) | 147585 | 6 | GGUUCCCAUCCAACAUGAAtt |
| POLDIP2 | polymerase (DNA-directed), delta interacting protein 2 | [26073](http://www.ncbi.nlm.nih.gov/entrez/query.fcgi?db=gene&cmd=Retrieve&dopt=Graphics&list_uids=26073) | 147586 | 11 | GCUUUAGGUAUGGAUUGAUtt |
| POLDIP2 | polymerase (DNA-directed), delta interacting protein 2 | [26073](http://www.ncbi.nlm.nih.gov/entrez/query.fcgi?db=gene&cmd=Retrieve&dopt=Graphics&list_uids=26073) | 147587 | 11 | CGUGAGGUUUGAUCAGUAAtt |
| RTRAF | RNA transcription, translation, and transport factor | [51637](http://www.ncbi.nlm.nih.gov/entrez/query.fcgi?db=gene&cmd=Retrieve&dopt=Graphics&list_uids=51637) | 23656 | 2 | GGGAAUUUAAGAAACAUCCtt |
| RTRAF | RNA transcription, translation, and transport factor | [51637](http://www.ncbi.nlm.nih.gov/entrez/query.fcgi?db=gene&cmd=Retrieve&dopt=Graphics&list_uids=51637) | 134783 | 8 | GGCAAUUAUUGCUGAUCCAtt |
| RTRAF | RNA transcription, translation, and transport factor | [51637](http://www.ncbi.nlm.nih.gov/entrez/query.fcgi?db=gene&cmd=Retrieve&dopt=Graphics&list_uids=51637) | 134784 | 8 | CCUACUUAGUACAGUUGGGtt |
| UPF1 | UPF1 regulator of nonsense transcripts homolog (yeast) | [5976](http://www.ncbi.nlm.nih.gov/entrez/query.fcgi?db=gene&cmd=Retrieve&dopt=Graphics&list_uids=5976) | 12197 | 2 | GGAAGAUGAAGAAGACACCtt |
| UPF1 | UPF1 regulator of nonsense transcripts homolog (yeast) | [5976](http://www.ncbi.nlm.nih.gov/entrez/query.fcgi?db=gene&cmd=Retrieve&dopt=Graphics&list_uids=5976) | 12290 | 4 | GGGCAAAAUGCAAAGAGGUtt |
| UPF1 | UPF1 regulator of nonsense transcripts homolog (yeast) | [5976](http://www.ncbi.nlm.nih.gov/entrez/query.fcgi?db=gene&cmd=Retrieve&dopt=Graphics&list_uids=5976) | 12379 | 24 | GGAAAAAAAACUUCGCAUUtt |
| YBX1 | Y box binding protein 1 | [4904](http://www.ncbi.nlm.nih.gov/entrez/query.fcgi?db=gene&cmd=Retrieve&dopt=Graphics&list_uids=4904) | 5109 | 2 | GGUUUUGGGAACAGUAAAAtt |
| YBX1 | Y box binding protein 1 | [4904](http://www.ncbi.nlm.nih.gov/entrez/query.fcgi?db=gene&cmd=Retrieve&dopt=Graphics&list_uids=4904) | 5291 | 3 | GGAAGAUGUAUUUGUACACtt |
| YBX1 | Y box binding protein 1 | [4904](http://www.ncbi.nlm.nih.gov/entrez/query.fcgi?db=gene&cmd=Retrieve&dopt=Graphics&list_uids=4904) | 115541 | 5 | GGCAGCAAAUGUUACAGGUtt |

**S2 Table. siRNAs.**
