# Supplementary material for: Elucidation of TRIM25 ubiquitination targets involved in diverse cellular and antiviral processes
Source: PLoS Pathog. 2022 Sep 6;18(9):e1010743. doi: 10.1371/journal.ppat.1010743 (PMC9481182; doi:10.1371/journal.ppat.1010743)
Supplement: S3 Table — (DOCX) [file ppat.1010743.s008.docx]

| **Target Gene** | **Name** | **Primer sequence (5'-3')** |
| --- | --- | --- |
| **CXorf56** | CXorf56 RT-qPCR F1 (EY-181) | CGGTCCCGTGTGATTGATG |
|  | CXorf56 RT-qPCR R1 (EY-182) | CCGCAGATACATAGTCTCCTCAT |
| **DNAJA1** | DNAJA1 RT-qPCR F1 (EY-183) | ACTGGAGCCAGGCGATATTAT |
|  | DNAJA1 RT-qPCR R1 (EY-184) | CTTCAACGAGCTGTATGTCCAT |
| **GLYR1** | GLYR1 RT-qPCR F1 (EY-185) | AGAAACCTCGCGGAAAGAAAT |
|  | GLYR1 RT-qPCR R1 (EY-186) | GACAGCATCTACCGCTTGC |
| **IFIT1** | IFIT1 RT-qPCR F1 (EY-134) | TTGATGACGATGAAATGCCTGA |
|  | IFIT1 RT-qPCR R1 (EY-135) | CAGGTCACCAGACTCCTCAC |
| **IFN-β** | IFN-β RT-qPCR F1 (EY-98) | GTCAGAGTGGAAATCCTAAG |
|  | IFN-β RT-qPCR R1 (EY-99) | ACAGCATCTGCTGGTTGAAG |
| **ISG15** | ISG15 RT-qPCR F1 (YD-09) | GGCTGGGAGCTGACGGTGAAG |
|  | ISG15 RT-qPCR R1 (YD-10) | GCTCCGCCCGCCAGGCTCTGT |
| **MOV10** | MOV10 RT-qPCR F1 (EY-151) | GGGCCAGTGTTTCGAGAGTTT |
|  | MOV10 RT-qPCR F1 (EY-152 | TCTTGGTGACGTAGGCCAGA |
| **MRPS26** | MRPS26 RT-qPCR F1 (EY-189) | CCAAATCCAAGATCGAGCGAG |
|  | MRPS26 RT-qPCR R1 (EY-190) | GGCGGTAGTGCTGGTAACG |
| **MRPS9** | MRPS9 RT-qPCR F1 (EY-191) | TGTAACCAGAGACGTGATTGGC |
|  | MRPS9 RT-qPCR R1 (EY-192) | TAGCAGCCGAATGAACTGCAT |
| **NCL** | NCL RT-qPCR F1 (EY-193) | GCACCTGGAAAACGAAAGAAGG |
|  | NCL RT-qPCR R1 (EY-194) | GAAAGCCGTAGTCGGTTCTGT |
| **NME1** | NME1 RT-qPCR F1 (EY-159) | AAGGAGATCGGCTTGTGGTTT |
|  | NME1 RT-qPCR R1 (EY-160) | CTGAGCACAGCTCGTGTAATC |
| **OAS2** | OAS2 RT-qPCR F1 (YD-11) | CGGTGTATGCCTGGGAACAGG |
|  | OAS2 RT-qPCR R1 (YD-12) | GGGTCAACTGGATCCAAGATTAC |
| **PABPC1** | PABPC1 RT-qPCR F1 (EY-195) | CAGGCTCACCTCACTAACCAG |
|  | PABPC1 RT-qPCR R1 (EY-196) | GGTAGGGGTTGATTACAGGGT |
| **PABPC4** | PABPC4 RT-qPCR F1 (EY-197) | TGGTAAGACCCTAAGTGTCAAGG |
|  | PABPC4 RT-qPCR R1 (EY-198) | TCCTCGTGTTTTTCGTAACTCAC |
| **POLDIP2** | POLDIP2 RT-qPCR F1 (EY-199) | CACCTCTCGTCCCGAAACC |
|  | POLDIP2 RT-qPCR R1 (EY-200) | CCATTCTGTTTTGGCACCTCAA |
| **RPS11** | RPS11 RT-qPCR F1 | GCCGAGACTATCTGCACTAC |
|  | RPS11 RT-qPCR R1 | ATGTCCAGCCTCAGAACTTC |
| **RTRAF** | RTRAF RT-qPCR F1 (EY-221) | TTCCGACGCAAGTTGACGG |
|  | RTRAF RT-qPCR R1 (EY-222) | CTGTCTTCAATCTTGTAGTGCCT |
| **UPF1** | UPF1 RT-qPCR F1 (EY-149) | CTGCAACGGACGTGGAAATAC |
|  | UPF1 RT-qPCR R1 (EY-150) | ACAGCCGCAGTTGTAGCAC |
| **YBX1** | YBX1 RT-qPCR F1 (EY-223) | GGGGACAAGAAGGTCATCGC |
|  | YBX1 RT-qPCR R1 (EY-224) | CGAAGGTACTTCCTGGGGTTA |

**S3 Table. RT-qPCR primers.**
